# Explainable AI uncovers novel EEG microstate candidate neurophysiological markers for autism spectrum disorder

**DOI:** 10.3389/fncom.2026.1763727

**Published:** 2026-02-04

**Authors:** Delna Kuriyakose, Gowsalya M.

**Affiliations:** Department of Mathematics, School of Advanced Sciences, Vellore Institute of Technology, Vellore, India

**Keywords:** autism spectrum disorder, biomarkers, EEG, explainable AI, microstates, multidomain features, temporal dynamics

## Abstract

**Background:**

Autism spectrum disorder (ASD) is a complex neurodevelopmental condition characterized by atypical brain connectivity and impaired cognitive flexibility. Electroencephalography (EEG) based microstate analysis provides insight into the rapid temporal dynamics of brain networks, offering potential biomarkers for ASD.

**Objective:**

This study proposes an interpretable classification framework for ASD diagnosis using multidomain microstate-informed features derived from EEG, integrating temporal, spectral, complexity-based, and higher-order metrics to comprehensively characterize brain dynamics.

**Methods:**

Resting state EEG data from 56 participants (28 with ASD and 28 neurotypical controls; age range: 18–68 years) from the publicly available Sheffield dataset were preprocessed and segmented into microstates using a data-driven clustering approach. From these microstate sequences, we extracted a rich set of features across four domains: (i) temporal, (ii) spectral, (iii) temporal complexity, and (iv) higher-order metrics. Multiple classifiers were evaluated using 10-fold cross-validation, with hyperparameter tuning via a randomized search.

**Results:**

Among all classifiers, XGBoost achieved the highest performance, with an accuracy of 80.87% when utilizing the complete multidomain feature set, significantly outperforming single domain models. Explainable AI analysis using SHapley Additive exPlanations (SHAP) identified the top 20 discriminative features, including fractional occupancy derivative for microstate 3, delta-band power in states 1 and 3, and mean inter-transition interval. Retraining XGBoost on these SHAP-selected features yielded 80.34% accuracy, confirming their robustness as potential biomarkers. Statistical validation via Mann–Whitney *U*-tests and effect size measures further established their significance.

**Conclusion:**

The findings from the study demonstrated that microstate-informed features capturing temporal instability, transition unpredictability, and spectral alterations serve as clinically relevant and interpretable candidate neurophysiological markers of ASD, offering translational potential for objective diagnosis, treatment monitoring, and personalized interventions.

## 1 Introduction

Autism spectrum disorder (ASD) is a complex neurodevelopmental condition that affects social interaction, communication, and behavior, with substantial heterogeneity in clinical presentation and underlying neurobiology (National Institute of Mental Health, 2024). Despite decades of research, ASD diagnosis still relies primarily on behavioral assessments, which, though informative, remain inherently subjective and variable across individuals and contexts. This subjectivity underscores the urgent need for objective, neurobiologically grounded biomarkers to support more consistent diagnosis and personalized intervention strategies.

Among the various neuroimaging modalities, electroencephalography (EEG) has emerged as a particularly promising tool for biomarker discovery due to its non-invasiveness, cost-effectiveness, and millisecond-level temporal resolution, enabling real-time monitoring of neural dynamics. Prior studies have shown that EEG captures subtle yet meaningful alterations in brain function associated with ASD, such as atypical oscillatory power, disrupted neural synchrony, and aberrant temporal organization of cortical activity (Coben et al., 2008; Bosl et al., 2011). These findings highlight EEG's potential to serve as a window into the fast-changing neural processes that underlie ASD.

Recent advances in machine learning (ML) and deep learning (DL) have further propelled EEG-based ASD research, enabling automated detection of diagnostic patterns that traditional methods can discern (Zhang et al., 2020; Cavus et al., 2021). Models trained on EEG data have successfully differentiated ASD from neurotypical (NT) individuals using features such as altered spectral power, connectivity, and event-related responses (Bosl et al., 2011; Duffy and Als, 2012; Sheikhani et al., 2012). Significantly, existing research has concentrated predominantly on infant and pediatric cohorts. Conversely, the present study analyzes EEG data from an adult ASD cohort, thereby addressing a comparatively underexplored developmental stage. Despite recent advances in ML for EEG-based autism classification, many current approaches suffer from significant limitations that hinder their clinical translation. Most ML models function as “black boxes,” offering high classification accuracy but providing little to no insight into why a decision was made, making it difficult for clinicians to trust or act upon these predictions in a medical setting (Doshi-Velez and Kim, 2017). This lack of interpretability is a critical barrier, as healthcare professionals require transparency into which brain regions, signal dynamics, or features are contributing to the diagnosis. Furthermore, many existing studies rely on single-domain EEG features, focusing solely on either spectral (e.g., frequency power) or temporal (e.g., signal variability) characteristics, thereby neglecting the rich interrelationships between domains such as spectral complexity, microstate transitions, or graph-theoretical dynamics (Fraschini et al., 2016; Craik et al., 2019). This fragmented approach may limit the discovery of more robust, generalizable biomarkers.

To address these challenges, we propose a novel microstate-informed multi-domain framework that integrates diverse EEG features to serve as robust biomarkers for ASD identification. Electroencephalographic (EEG) microstate analysis is a promising framework for discovering objective biomarkers in neurodevelopmental disorders, including ASD (Michel and Koenig, 2018; Khanna et al., 2015). EEG microstates are short-lived, quasi-stable spatial patterns in scalp potential topographies that persist for approximately 60–120 ms. Microstate analysis captures the spatiotemporal evolution of brain activity, linking transient topographies to underlying cognitive and neural processes (Britz et al., 2010; Lehmann et al., 2005). Unlike traditional EEG features that focus on static spectral or temporal characteristics, microstate analysis enables a dynamic assessment of brain activity through sequence-like representations of functional states (Koenig et al., 2002).

To maximize the diagnostic potential of microstate analysis, our framework integrates microstate-derived features across four complementary domains: temporal, spectral, complexity, and graph-theoretical metrics to construct a holistic representation of brain dynamics. Each domain captures unique neurophysiological aspects of brain function: temporal features reflect state stability, spectral features relate to neural oscillatory behavior, complexity metrics quantify the irregularity and structure of sequences, and graph-based features model transitions between states as dynamic networks (Van De Ville et al., 2010; Babiloni et al., 2016; Ahmadlou and Adeli, 2010). Integrating these multi-domain microstate-informed features allows for a more holistic and nuanced characterization of atypical brain function in ASD, moving beyond the limitations of single-domain EEG approaches (D'Croz-Baron et al., 2019).

To enhance interpretability and foster clinical trust, our approach incorporates SHAP (SHapley Additive exPlanations), a widely used explainability method rooted in cooperative game theory (Lundberg and Lee, 2017). SHAP assigns each feature an importance value for individual model predictions by evaluating the marginal contribution of that feature across all possible combinations of inputs. Unlike traditional feature importance methods, SHAP provides both global (overall importance) and local (subject-specific) insights, making it especially suitable for uncovering the neurophysiological relevance of EEG-derived biomarkers in ASD. By visualizing how specific EEG features influence classification outcomes, SHAP enables transparent decision-making and helps bridge the gap between complex ML models and clinical interpretability. To further support the validity of these features, we applied rigorous statistical testing, including non-parametric methods such as the Mann–Whitney *U*-test (Mann and Whitney, 1947), and calculated effect sizes (e.g., Cohen's *d*, Cliff's delta) (Cohen, 1988; Romano et al., 2006) to quantify group differences. Ultimately, this research seeks to identify interpretable, microstate-informed EEG biomarkers that capture the disrupted temporal stability, spectral dynamics, and large-scale network coordination characteristic of ASD. By integrating data-driven ML with physiologically grounded microstate analysis, the study advances toward the development of objective, reliable, and clinically translatable diagnostic tools for ASD. We hypothesize that a multi-domain characterization of EEG microstates, encompassing temporal, spectral, complexity, and network-level features, when interpreted through an explainable AI framework, will yield robust, neurobiologically meaningful biomarkers capable of distinguishing ASD from neurotypical controls.

## 2 Related study

Autism spectrum disorder (ASD) is currently diagnosed based on behavioral assessments such as difficulties in social communication, restricted interests, and repetitive behaviors. While these criteria are clinically validated, they are also subjective and highly dependent on clinical expertise. They may result in delayed or inconsistent diagnoses, particularly in young children or under-resourced settings (Lord et al., 2020). In response to these limitations, researchers have increasingly turned their attention to biological markers that could provide objective support for diagnosis and prognosis. Several types of biomarkers have been explored in the ASD literature, including genetic factors (e.g., rare de novo mutations, copy number variants), metabolic signatures (e.g., amino acid and mitochondrial abnormalities), and neuroimaging features derived from structural and functional MRI (Endres et al., 2019; Thapar, 2017; Klin, 2018; Frye et al., 2022).

Although these biomarkers have yielded valuable insights into ASD pathophysiology and heterogeneity, they also come with important limitations. Genetic and metabolic markers often lack real-time functional information, and neuroimaging techniques like fMRI, while spatially precise, are expensive, non-portable, and not feasible for repeated or widespread screening.

Conversely, EEG offers a compelling alternative: it is non-invasive, cost-effective, portable, and provides millisecond-level temporal resolution, making it well-suited for capturing fast-changing neural dynamics that may underlie ASD-specific processing differences (Mendoza-Palechor et al., 2019). Given these strengths, EEG is increasingly viewed as a promising modality for discovering robust, interpretable, and clinically applicable biomarkers of ASD.

Historically, EEG has played a pivotal role in elucidating atypical neural dynamics associated with ASD. Traditional analyses have focused on time-domain features, such as fluctuations in event-related potentials (ERPs), and studies have reported delayed or diminished responses in ASD during cognitive and sensory tasks (Jeste and Nelson, 2009). Spectral domain investigations have uncovered abnormal oscillatory activity, including increased delta and theta power and reduced alpha and beta-band synchronization, reflecting potential impairments in cortical excitability and information processing (Wang et al., 2013). Moreover, connectivity-based EEG approaches, such as coherence and phase-locking value (PLV), have revealed disrupted functional communication across brain regions, particularly in fronto-temporal and fronto-parietal networks (Coben et al., 2008). These findings provide valuable neurophysiological signatures that correlate with ASD symptomatology, including deficits in social cognition, attention, and sensory integration.

Despite these advancements, the utility of EEG-derived features as clinically viable biomarkers remains limited. A primary concern is that many of these features, when used in isolation, lack robustness and generalizability across individuals and datasets (Bosl et al., 2011). Variability in EEG acquisition protocols, subject heterogeneity, and analytical pipelines often lead to inconsistent or non-replicable findings. Furthermore, most studies rely on black-box ML models such as support vector machines (SVM), deep neural networks, or ensemble classifiers, that, while potentially achieving high accuracy, offer limited insight into which neurophysiological features drive predictions. This lack of interpretability impedes clinical adoption, which requires explainability and transparency. As emphasized by Gangaraju and Yogisha (2025), the field urgently requires integrative, explainable, and statistically rigorous frameworks to harness EEG for ASD biomarker discovery.

EEG microstates have emerged as a promising avenue for uncovering high-level, temporally resolved biomarkers of brain function. Unlike traditional time- or frequency-domain features, microstates represent brief, quasi-stable topographical patterns that last approximately 60–120 ms and are thought to reflect the coordinated activity of large-scale neural networks across the brain. Often referred to as the “atoms of thought” (Lehmann et al., 2005), these microstates capture rapid spatiotemporal dynamics associated with distinct cognitive and perceptual processes (Michel and Koenig, 2018). Microstate analysis has demonstrated clinical relevance in a variety of neuropsychiatric conditions, such as schizophrenia (Kindler et al., 2011), depression (Strik et al., 1995), and epilepsy (Javed et al., 2019), offering a functional lens into altered brain dynamics.

However, despite its utility, the application of microstate analysis in ASD remains limited. Only a few exploratory studies have examined microstate alterations in ASD, typically focusing on global properties such as duration or occurrence (D'Croz-Baron et al., 2019; Takarae et al., 2022; Das et al., 2022). This underscores a critical research gap: the need for comprehensive microstate-informed analysis in ASD that spans multiple domains, including temporal, spectral, complexity, and network-based, to enable interpretable and clinically relevant biomarker discovery.

While EEG microstate analysis has gained attention as a promising tool for probing altered neural dynamics in ASD, most existing studies remain limited in scope. Several studies have focused on temporal features such as average microstate duration and occurrence rate to detect group-level differences between ASD and neurotypical populations (Chaudhary et al., 2021; Bochet et al., 2021). Others have investigated transition probabilities between states, aiming to characterize disrupted microstate syntax in ASD (Chen et al., 2023; Ma et al., 2025). However, these efforts largely operate within a single feature domain and often rely on descriptive statistics or classifiers. Crucially, there is a lack of integrated pipelines that combine multi-domain features, including spectral power, entropy-based complexity metrics, and graph-theoretic measures of microstate transitions to yield a more comprehensive neurophysiological fingerprint of ASD. Furthermore, few studies have incorporated model interpretability frameworks such as SHapley Additive exPlanations (SHAP) (Zeraati and Davoodi, 2025; Jeon et al., 2024), limiting clinical insight and trust in classification decisions. This highlights a significant research gap and the need for explainable, multi-domain microstate-informed models for ASD biomarker discovery.

To bridge this gap, we propose a novel multi-domain analytical pipeline that extracts and integrates features from four distinct but complementary domains: (i) temporal dynamics, such as microstate duration and transition entropy; (ii) spectral power computed within microstate defined segments; (iii) complexity metrics, including permutation entropy and fractal dimension to capture non-linear neural variability; and (iv) graph theoretical measures derived from microstate transition matrices, offering insights into the topological structure of brain dynamics. Importantly, our method uniquely integrates SHAP-based interpretability to determine feature importance at both global and individual levels, complemented by robust statistical validation through nonparametric tests and effect-size analysis. This design not only addresses the limitations of black-box ML models but also provides interpretable, statistically validated biomarkers that may hold translational relevance for ASD diagnosis. The following section describes this methodology in detail.

## 3 Materials and methods

**Figure 1:**
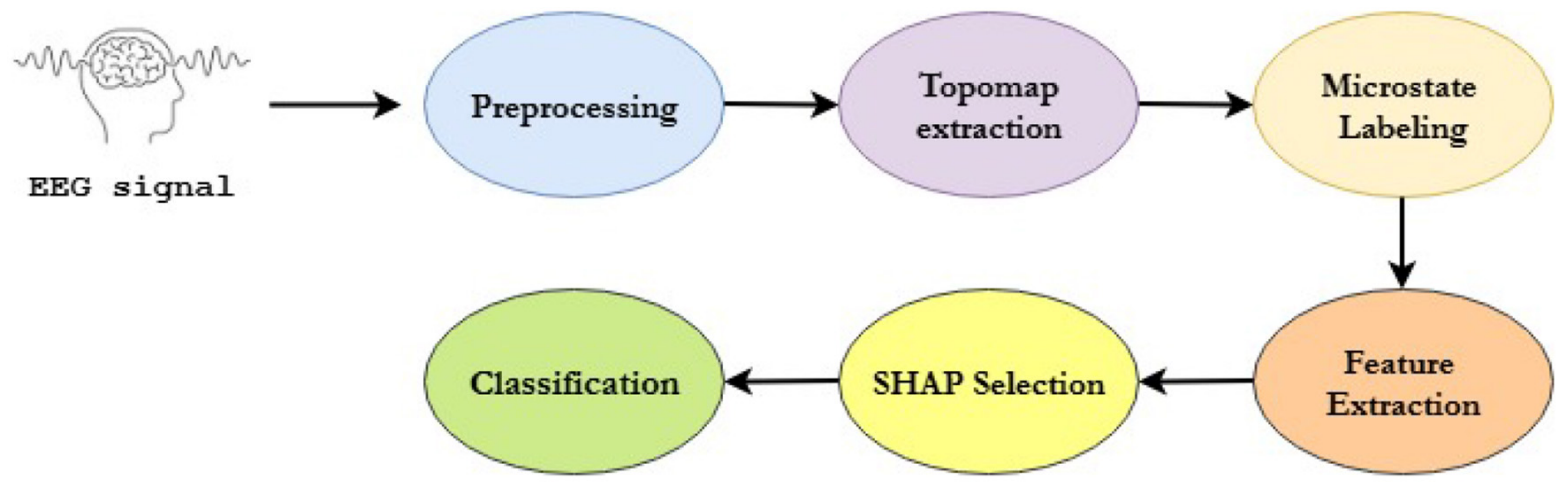
Overview of methodology.

In this section, we present the materials and methods used for microstate segmentation, a neurophysiologically interpretable approach to EEG-based diagnosis of ASD. The primary objective of this study is to harness microstate analysis to uncover temporal brain dynamics that distinguish individuals with ASD from neurotypical controls. Leveraging the publicly available Sheffield EEG dataset, we implement a comprehensive pipeline, as shown in Figure 1, encompassing data preprocessing, graph construction, multi-domain feature extraction, and classification. Our goal is to achieve both diagnostic accuracy and model interpretability, addressing current limitations in EEG-based ASD research. The methodology is structured into the following subsections: (1) Dataset Description, (2) Data Preprocessing, (3) Microstate Segmentation, (4) Multi-domain Feature Extraction, and (5) Classification and Model Evaluation.

### 3.1 Dataset description

The study utilized resting-state EEG data from the publicly available Sheffield Autism EEG Dataset (Milne, 2021), which comprises electrophysiological recordings from 28 individuals with a diagnosis of an autism spectrum condition and 28 neurotypical controls aged between 18 and 68 years. The experimental paradigm comprised a 2.5-minutes (150-seconds) resting-state period with eyes closed. EEG data were acquired using a Biosemi Active two EEG system. The original recordings have been converted to .set and .fdt files via EEGLAB. Each recording has a corresponding a .fdt and a .set file; the .fdt file contains the data, the .set file contains information about the parameters of the recording. Ethical approval for data collection and data sharing was given by the Health Research Authority [IRAS ID = 212171].

The dataset used in this study has been previously analyzed in several published works investigating EEG alterations in ASD. For example, Milne et al. (2019) examined atypical resting-state EEG patterns in ASD using both categorical and dimensional analytical frameworks, highlighting differences in oscillatory activity and neural organization. More recently, Dickinson et al. (2022) explored electrophysiological signatures of brain aging in individuals with ASD using the same cohort, providing insights into age-related changes in neural dynamics. Referencing these studies provides important context regarding participant characteristics, recording protocols, and previously reported EEG features, and situates the present work within an established body of literature using this dataset.

### 3.2 Data preprocessing

Preprocessing plays a pivotal role in ensuring the quality and reliability of EEG data before advanced analysis. In this study, EEG preprocessing was performed using the EEGLAB toolbox in MATLAB, a widely used platform for electrophysiological data analysis. Continuous EEG signals were first band-pass filtered between 1 and 45 Hz using a zero-phase finite impulse response (FIR) filter to remove slow drifts and high-frequency noise while preserving frequency components relevant to cognitive and neural processes. A notch filter at 50 Hz was applied to suppress power-line interference. To enhance signal consistency across electrodes and facilitate inter-channel comparisons, the signals were re-referenced to the common average, a standard practice in EEG preprocessing. Subsequently, the data were resampled to 128 Hz, providing an optimal balance between temporal resolution and computational efficiency for downstream microstate and ML analyses.

Artifact handling was performed using an automated epoch-based rejection strategy. The continuous EEG signals were segmented into 2-s epochs with 50% overlap, enabling fine-grained temporal characterization while preserving continuity across segments. Epochs containing physiological artifacts such as eye blinks, muscle activity, or excessive noise were automatically identified using predefined amplitude and variance thresholds and excluded from further analysis. No manual artifact rejection was performed, ensuring objectivity and reproducibility of the preprocessing pipeline.

In the dataset, different subjects had varying numbers and configurations of EEG channels. This heterogeneity necessitated a standardization step to ensure uniformity across subjects, which is essential for consistent and comparable downstream analyses. Standardization is a vital preprocessing step in multi-subject EEG analysis to ensure structural consistency across datasets. In this study, standardization was accomplished by first examining the number and names of EEG channels for each subject. Based on this, the most frequently occurring channel configuration across the dataset was identified and selected as the reference standard. This reference layout represents the optimal set of channels that maximizes coverage across all subjects while enabling meaningful cross-subject comparison.

To conform each subject's data to this reference, a systematic alignment procedure was applied. For subjects with missing channels, those absent electrodes were artificially appended with zero-valued time series, effectively creating flat-line signals that serve as neutral placeholders without introducing noise. Conversely, any additional channels present in a subject's recording but not included in the standard reference were excluded to prevent structural mismatch. Through this approach, all EEG recordings were harmonized to exhibit (i) the same number of channels (*n* = 54), (ii) standardized channel names, and (iii) a consistent channel order.

This standardization protocol is crucial for downstream analyses such as topographical mapping, feature extraction, and ML classification that rely on spatial and anatomical consistency across EEG data. By ensuring uniformity in channel structure, the framework enhances the interpretability and reproducibility of inter-subject analyses, particularly for biomarker discovery for clinical applications like ASD diagnosis.

### 3.3 Microstate segmentation

EEG microstate segmentation is initiated by identifying Global Field Power (GFP) peaks, which serve as anchor points for extracting stable topographic maps. The proposed procedure begins by detecting Global Field Power (GFP) peaks and extracting topographic maps for each EEG epoch. GFP is defined as the standard deviation of the EEG signal across all channels at each time point, providing a measure of the overall strength and synchrony of brain activity. In this context, GFP highlights moments of high neural variability, which are hypothesized to correspond to transitions between distinct functional brain states. Global Field Power (GFP) at time point *t* is defined as the standard deviation of the EEG signal across all electrodes (channels), capturing the spatial variability of brain activity at that moment, and it is expressed as:


$$\begin{array}{l} \text{GFP}\left(t\right)=\sqrt{\frac{1}{N}\overset{N}{\underset{i=1}{\sum }}{\left(V_{i}\left(t\right)-\bar{V}\left(t\right)\right)}^{2}} \end{array}$$ (1)

where:

- *N* is the total number of EEG channels (e.g., *N* = 54),
- *V*_*i*_(*t*) is the voltage at channel *i* at time *t*,
- $\bar{V}\left(t\right)$ is the average voltage across all channels at time *t*, given by:

$$\bar{V}\left(t\right)=\frac{1}{N}\overset{N}{\underset{i=1}{\sum }}V_{i}\left(t\right)$$

- GFP(*t*) reflects the spatial standard deviation of scalp potentials at time *t*.

High GFP values indicate time points with strong spatial activation differences across the scalp and are used to identify peaks corresponding to transient and stable topographic brain states (microstates).

Peaks in the GFP signal representing these high-variance time points are identified, and the corresponding EEG topographic maps, reflecting the spatial distribution of brain activity across the scalp, are extracted. Each topography is represented as a 54-dimensional vector, corresponding to the number of EEG channels. These vectors are then aggregated across all epochs and subjects to form a matrix of spatial patterns, which serves as input for microstate clustering.

The next phase involves microstate template learning through K-Means clustering. In this step, all topographic maps extracted at GFP peaks across epochs and subjects are first concatenated into a single matrix and subsequently normalized to ensure each map has a unit norm. This normalization ensures that clustering is driven by spatial configuration rather than amplitude differences. For visualization, the four microstates are labeled alphabetically (A–D); however, throughout the analysis and feature extraction pipeline, these same microstates are indexed numerically as state 0, state 1, state 2, and state 3, respectively.

K-Means clustering aims to partition a set of *N* data points {**x**_1_, **x**_2_, …, **x**_*N*_} into *K* distinct clusters by minimizing the within-cluster variance. The objective function is defined as:


$$\begin{array}{l} \underset{{\left\{C_{k}\right\}}_{k=1}^{K}}{\arg\min}\overset{K}{\underset{k=1}{\sum }}\underset{{\text{x}}_{i}\in C_{k}}{\sum }\|{\text{x}}_{i}-\mu_{k}{\|}^{2} \end{array}$$ (2)

where:

- *N* is the total number of data points (e.g., GFP-peak topographies).
- *K* is the number of clusters (i.e., microstates), chosen a priori.
- ${\text{x}}_{i}\in {\mathbb{R}}^{D}$ is the *i*-th data point (e.g., a normalized topography vector of *D* = 54 EEG channels).
- $C_{k}$ is the set of data points assigned to cluster *k*.
- ***μ***_*k*_ is the centroid (mean) of cluster *k*.
- ‖·‖ denotes the Euclidean norm (i.e., squared distance).

The algorithm iteratively updates:

1. Cluster assignments: assign each point **x**_*i*_ to the nearest centroid.
2. Centroid update: recompute each ***μ***_*k*_ as the mean of points in $C_{k}$.

This continues until convergence, typically when assignments no longer change, or the decrease in the objective function falls below a threshold.

**Figure 2:**
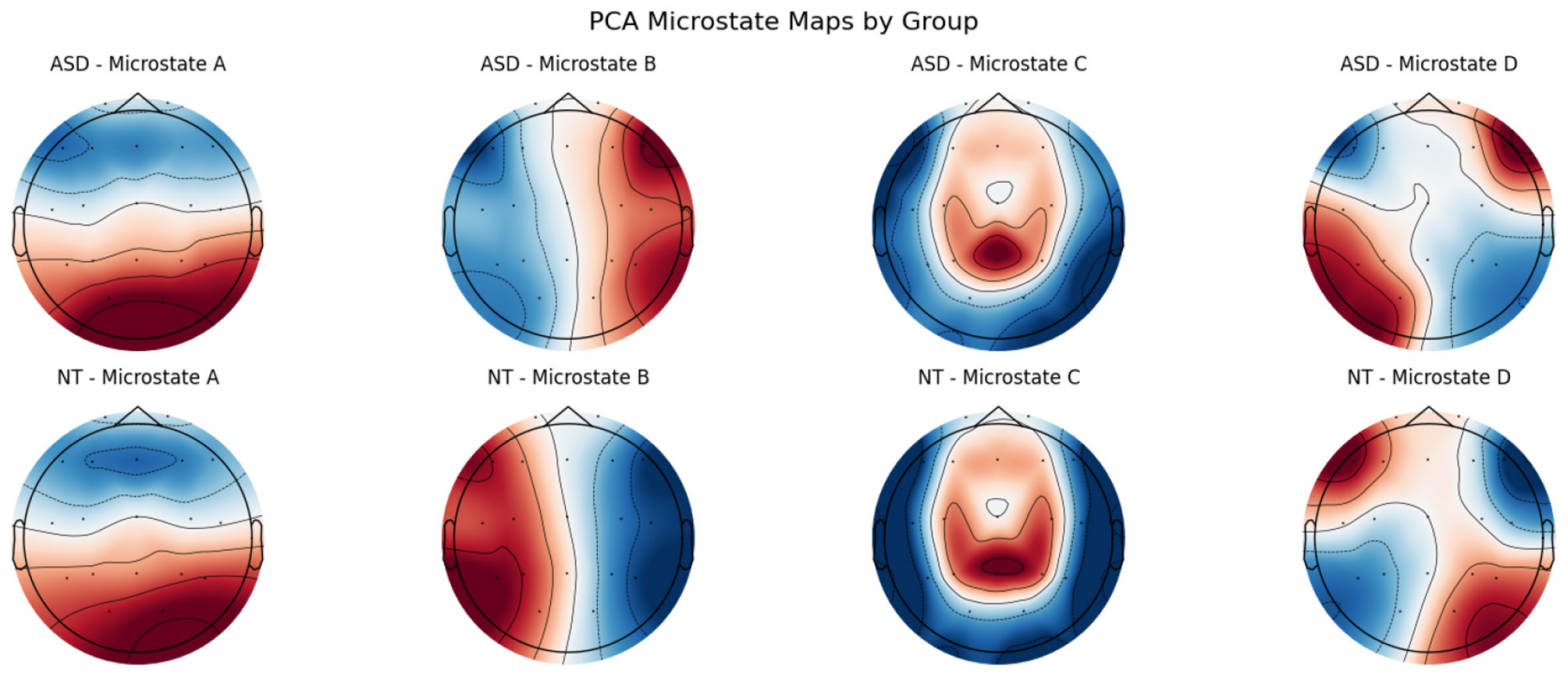
Topographic scalp maps of the four dominant EEG microstates extracted via PCA on GFP peaks for both ASD and NT groups. Microstates are labeled alphabetically **(A–D)** for visualization, corresponding to numerical indices used in analysis as follows: A = state 0, B = state 1, C = state 2, and D = state 3.

The K-Means algorithm is then applied to group these normalized topographies into a pre-defined number, which is 4, based on prior literature and empirical validation (Lehmann et al., 1987). Each resulting cluster represents a distinct microstate class, with the corresponding cluster centroid capturing the prototypical spatial pattern or template of that microstate. These centroids are stored as the canonical microstate maps and serve as reference topographies for subsequent segmentation. The K-Means clustering model yields four cluster centroids, stored in an array of shape (4, 54), where each row corresponds to one of the four learned microstates and each column represents an EEG channel. These centroids serve as spatial templates that capture the prototypical topographic patterns most representative of the GFP-peak EEG topographies across all subjects, as shown in Figure 2. During the segmentation stage, these microstate templates are used to assign a label to each time point in the EEG data by computing spatial similarity between the current EEG topography and each of the four templates, thereby determining the most likely microstate at each time point.

**Figure 3:**
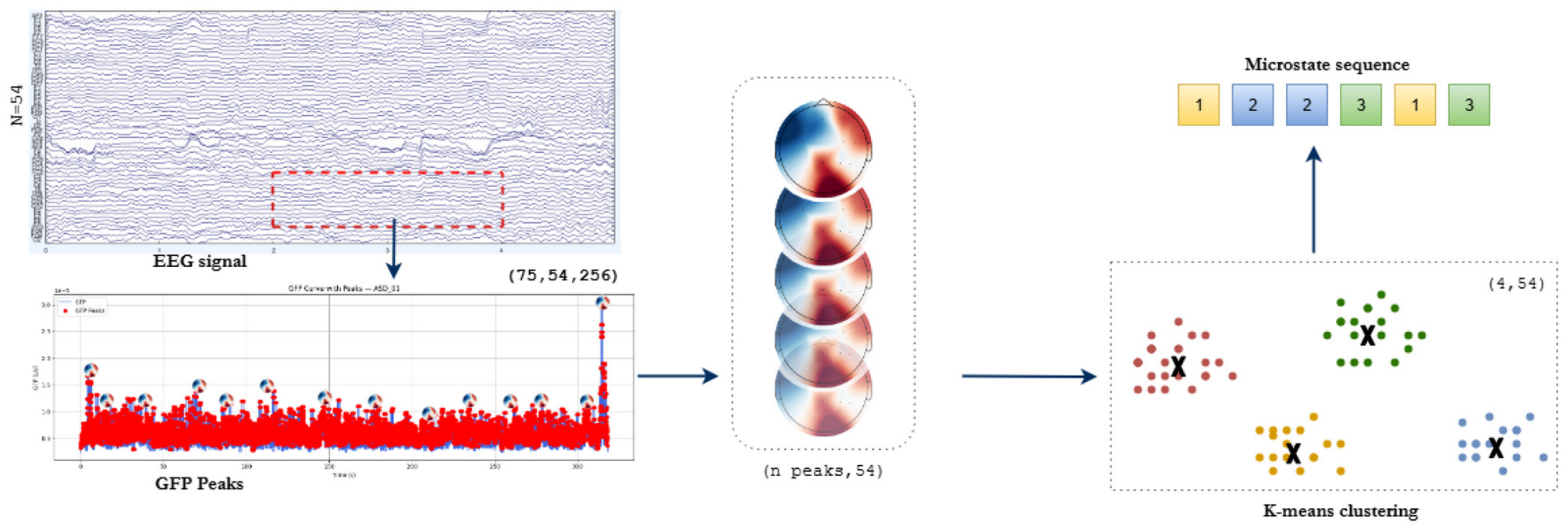
Workflow of EEG microstate segmentation.

Following the identification of microstate templates, the subsequent stage involves segmenting the EEG signal into discrete microstate sequences by assigning a microstate label to each time point based on spatial similarity. For each epoch of EEG data, the spatial pattern at every individual time point, represented as a vector across all EEG channels, is first normalized to unit length. This normalization ensures that the subsequent similarity computations are not biased by amplitude differences across time points. Once normalized, each time point's spatial pattern is compared to all four microstate templates using spatial correlation, operationalized as the dot product between the normalized EEG vector and each microstate centroid. This yields a correlation value for each template, reflecting how well the current spatial pattern matches each microstate. The time point is then labeled with the microstate corresponding to the highest correlation, effectively assigning the most likely underlying functional brain state at that moment. This process produces a sequence of microstate labels for each epoch, capturing the temporal dynamics of the brain's large-scale functional configurations, as depicted in Figure 3.

### 3.4 Multi-domain feature extraction

As the next stage of analysis, we extract a comprehensive set of features derived from the EEG microstate sequences, encompassing four distinct domains: microstate temporal, microstate spectral, microstate temporal complexity, and microstate higher-order transition features. This multidimensional feature extraction framework enables the integration of diverse neurophysiological characteristics ranging from time-based metrics to spectral dynamics, entropy-based complexity, and higher-order transition statistics. By grounding these features in microstate-informed representations of brain activity, our approach provides a more holistic and interpretable characterization of neural dynamics, facilitating robust and clinically meaningful classification of ASD.

#### 3.4.1 Microstate temporal features

Here we extract a diverse set of features that capture key aspects of microstate dynamics, including time spent in each microstate, switching behavior between microstates, transition probabilities, recurrence and determinism of state sequences, and entropy-based measures of temporal complexity. These features collectively reflect both the stability and variability of brain state transitions, offering valuable insights into the underlying neural organization and its potential alterations in ASD.

Let a subject's microstate sequence over time be denoted as:


$$S=\left[s_{1},s_{2},\ldots ,s_{T}\right],\;s_{t}\in \left\{1,2,\ldots ,K\right\}$$

where *T* is the total number of time points and *K* is the number of unique microstates (e.g., *K* = 4 in this study).

1. Mean duration of state *k*: reflects the average time the brain remains in microstate *k* before switching, capturing the stability of that functional configuration.
$$D_{k}=\frac{1}{N_{k}}\overset{N_{k}}{\underset{i=1}{\sum }}d_{i}^{\left(k\right)}$$
  where $d_{i}^{\left(k\right)}$ is the duration (in samples or timepoints) of the *i*^th^ uninterrupted segment of state *k*, and *N*_*k*_ is the total number of such segments.
2. Coverage (fractional occupancy) of state *k*: indicates the proportion of the total recording duration spent in state *k*, reflecting its dominance in brain activity.
$$C_{k}=\frac{1}{T}\overset{T}{\underset{t=1}{\sum }}\delta \left(s_{t}=k\right)$$
  where δ(·) is the indicator function.
3. Occurrence rate of state *k*: represents how frequently microstate *k* is activated throughout the EEG recording.
$$O_{k}=\frac{N_{k}}{T}$$
  where *N*_*k*_ is the number of transitions into state *k*.
4. Dwell entropy of state *k*: measures the variability in how long the brain stays in state *k*. Higher entropy suggests irregular durations and less predictability.
$$H_{k}=-\overset{N_{k}}{\underset{i=1}{\sum }}p_{i}^{\left(k\right)}\log p_{i}^{\left(k\right)},\;p_{i}^{\left(k\right)}=\frac{d_{i}^{\left(k\right)}}{\overset{N_{k}}{\underset{j=1}{\sum }}d_{j}^{\left(k\right)}}$$
5. Switching rate (SR): captures how often the brain transitions between different microstates, reflecting neural flexibility or instability.
$$SR=\frac{1}{T-1}\overset{T-1}{\underset{t=1}{\sum }}\delta \left(s_{t}\neq s_{t+1}\right)$$
6. Transition probability from state *i* to *j*: Models the dynamic switching behavior of brain states as a first-order Markov process.
$$P_{i\to j}=\frac{N_{i\to j}}{\overset{K}{\underset{j^{\prime }=1}{\sum }}N_{i\to j^{\prime }}}$$
  where *N*_*i*→*j*_ is the count of direct transitions from state *i* to *j*.
7. Recurrence rate (RR): indicates how frequently the same microstates reappear in the sequence, reflecting recurrent patterns in neural dynamics.
$$RR=\frac{1}{T^{2}}\overset{T}{\underset{t=1}{\sum }}\overset{T}{\underset{t^{\prime }=1}{\sum }}\delta \left(s_{t}=s_{t^{\prime }}\right)$$
8. Determinism (DET): quantifies the predictability of transitions by measuring the proportion of recurring, deterministic patterns in the sequence.
$$DET=\frac{\overset{L}{\underset{l=l_{\min}}{\sum }}l\cdot P\left(l\right)}{\overset{T}{\underset{i=1}{\sum }}\overset{T}{\underset{j=1}{\sum }}R_{i,j}}$$
  where *P*(*l*) is the number of diagonal lines of length *l* in the recurrence matrix *R*, and *l*_min_ is the minimum line length (e.g., *l*_min_ = 2).

To summarize, the microstate temporal feature extraction yields 35 features per epoch. This includes four features each for mean duration, coverage, occurrence rate, and dwell entropy across the 4 microstates (4 × 4 = 16), a 16-element transition probability matrix (4 × 4), along with 1 feature each for switching rate, recurrence rate, and determinism. Together, these features offer a comprehensive temporal characterization of microstate dynamics in the EEG data.

#### 3.4.2 Microstate-spectral features

For each identified microstate, we extracted state-specific average EEG power within standard frequency bands to capture its spectral characteristics. The analysis is grounded in canonical EEG frequency ranges: Delta (1–4 Hz), typically associated with deep sleep and pathological slowing; Theta (4–8 Hz), linked to memory and drowsiness; Alpha (8–13 Hz), reflecting relaxed wakefulness; Beta (13–30 Hz), associated with active thinking and focus; and Gamma (30–45 Hz), related to sensory integration and attention.

To perform this, we first identify time points corresponding to each microstate and exclude segments in which the duration of a microstate is less than 1 s, ensuring reliable spectral estimation. We then apply Welch's method to compute the Power Spectral Density (PSD) for each EEG channel at those selected timepoints, yielding a matrix of shape (54, n_frequencies). For each frequency band, we calculated the mean power across the corresponding frequency range and subsequently averaged these values across all channels. This results in a compact, interpretable spectral profile for each microstate.

For each microstate *k* ∈ {1, 2, 3, 4} the spectral power in each band is defined as:


$$\begin{array}{l} P_{\delta }^{\left(k\right)}=\frac{1}{N_{c}}\overset{N_{c}}{\underset{c=1}{\sum }}\int_{1}^{4}{\text{PSD}}_{c}^{\left(k\right)}\left(f\right)df\\ P_{\theta }^{\left(k\right)}=\frac{1}{N_{c}}\overset{N_{c}}{\underset{c=1}{\sum }}\int_{4}^{8}{\text{PSD}}_{c}^{\left(k\right)}\left(f\right)df\\ P_{\alpha }^{\left(k\right)}=\frac{1}{N_{c}}\overset{N_{c}}{\underset{c=1}{\sum }}\int_{8}^{13}{\text{PSD}}_{c}^{\left(k\right)}\left(f\right)df\\ P_{\beta }^{\left(k\right)}=\frac{1}{N_{c}}\overset{N_{c}}{\underset{c=1}{\sum }}\int_{13}^{30}{\text{PSD}}_{c}^{\left(k\right)}\left(f\right)df\\ P_{\gamma }^{\left(k\right)}=\frac{1}{N_{c}}\overset{N_{c}}{\underset{c=1}{\sum }}\int_{30}^{45}{\text{PSD}}_{c}^{\left(k\right)}\left(f\right)df \end{array}$$

where:

- $P_{\delta }^{\left(k\right)}$, $P_{\theta }^{\left(k\right)}$, $P_{\alpha }^{\left(k\right)}$, $P_{\beta }^{\left(k\right)}$, $P_{\gamma }^{\left(k\right)}$: Average delta, theta, alpha, beta, and gamma band power for microstate *k*.
- *N*_*c*_: Total number of EEG channels.
- ${\text{PSD}}_{c}^{\left(k\right)}\left(f\right)$: Power Spectral Density of channel *c* during timepoints labeled as microstate *k*, as a function of frequency *f*.
- The integration limits represent the standard frequency bands: delta (1–4 Hz), theta (4–8 Hz), alpha (8–13 Hz), beta (13–30 Hz), and gamma (30–45 Hz).

In total, the spectral feature extraction yields 20 features per subject, derived from computing average power across five canonical frequency bands (delta, theta, alpha, beta, gamma) for each of the four microstates. These features provide a frequency-specific spectral signature of brain activity associated with distinct microstate configurations.

#### 3.4.3 Temporal complexity features

Here, the subject's microstate sequence is first loaded. If it is organized on a per-epoch basis (i.e., in a 2D format), it is flattened into a continuous 1D sequence to preserve temporal continuity. Subsequently, a series of temporal complexity features is computed over the entire microstate sequence, enabling quantification of the structural and dynamical characteristics of microstate transitions.

To characterize the dynamic structure of the microstate sequences, we computed several nonlinear temporal complexity metrics. These features quantify the irregularity, self-similarity, and information content of the symbolic sequence of microstates. We extracted the following features:

1. Sample entropy : sample entropy quantifies the unpredictability or regularity of a time series by measuring the probability that patterns of length *m* that match will also match at length *m*+1.
$$\begin{array}{l} \text{SampEn}\left(m,r,N\right)=-\ln\;\left(\frac{A}{B}\right) \end{array}$$ (3)
  - *m*—embedding dimension (pattern length)
  - *r*—tolerance for accepting matches (as a proportion of the standard deviation)
  - *N*—length of the time series
  - *A*—number of matches of length *m*+1
  - *B*—Number of matches of length *m*
2. Permutation entropy: permutation entropy measures the complexity of a time series based on the frequency of ordinal patterns. Higher values indicate more randomness in the ordering of the data.
$$\begin{array}{l} \text{PermEn}\left(m\right)=-\overset{k}{\underset{i=1}{\sum }}p_{i}\log p_{i} \end{array}$$ (4)
  - *m*—embedding dimension (number of points in each pattern)
  - *p*_*i*_—probability of the *i*th permutation
  - *k* = *m*!—total number of possible patterns
3. Detrended fluctuation analysis (DFA): DFA quantifies the presence of long-range correlations in a time series by evaluating how fluctuations scale with time window size.
$$\begin{array}{l} F\left(n\right)=\sqrt{\frac{1}{N}\overset{N}{\underset{i=1}{\sum }}{\left[y\left(i\right)-y_{n}\left(i\right)\right]}^{2}}\;\text{and}\;F\left(n\right)\propto n^{\alpha } \end{array}$$ (5)
  - *y*(*i*)—integrated signal
  - *y*_*n*_(*i*)—local trend in segment of size *n*
  - α—Scaling exponent indicating correlation strength
  A value of α = 0.5 indicates uncorrelated (white noise), α > 0.5 indicates long-range correlation, and α < 0.5 suggests anti-persistence.
4. Lempel-Ziv complexity (LZC): LZC estimates the number of distinct patterns in a sequence. It reflects the sequence's compressibility–higher values indicate higher randomness and lower redundancy.
$$\begin{array}{l} \text{LZC}\left(S\right)=\frac{c\left(S\right)}{n/\log_{2}n} \end{array}$$ (6)
  - *S*—input binary or symbolic sequence
  - *c*(*S*)—number of distinct substrings (complexity counter)
  - *n*—length of the sequence
5. Hurst exponent (H): the Hurst exponent evaluates the tendency of a time series to regress to the mean or to cluster in a particular direction, thereby measuring long-term memory.
$$\begin{array}{l} E\left[\frac{R\left(n\right)}{S\left(n\right)}\right]\propto n^{H} \end{array}$$ (7)
  - *R*(*n*)—range of cumulative deviations
  - *S*(*n*)—standard deviation within window of size *n*
  - *H*—Hurst exponent; *H* > 0.5 indicates persistent behavior, *H* < 0.5 anti-persistent, *H* = 0.5 random walk

#### 3.4.4 Higher-order microstate sequence features

To capture higher-order temporal dynamics of EEG microstate sequences, we extract n-gram-based features, with a focus on 3-gram entropy. This measure evaluates the complexity and unpredictability of short sequences of three consecutive microstates, offering insight into structured vs. random transition patterns. Before computing the n-gram entropy, we remap the microstate labels to ensure consistent indexing. Specifically, all unique state labels within a sequence are reassigned to contiguous integers starting from 0. This preprocessing step standardizes the input, which is particularly necessary when some subjects do not exhibit all predefined microstates (e.g., if a sequence includes only states 2 and 3, they are remapped to [0, 1]).

Let a symbolic microstate sequence be denoted as:


$$S=\left[s_{1},s_{2},\ldots ,s_{T}\right],\;s_{t}\in \left\{1,2,\ldots ,K\right\}$$

where *T* is the number of time points, and *K* is the total number of distinct microstates (typically *K* = 4).

We extract the following higher-order features from the microstate sequence:

1. Transition entropy: quantifies the uncertainty in the transition dynamics; higher values indicate more stochastic switching.
$$H_{\text{trans}}=-\overset{K}{\underset{i=1}{\sum }}\overset{K}{\underset{j=1}{\sum }}P_{ij}\log P_{ij}$$
  where *P*_*ij*_ is the empirical probability of transitioning from microstate *i* to *j*.
2. Mean inter-transition interval: captures short-term temporal complexity in the state sequence.
$$\mu_{\text{ITI}}=\frac{1}{N-1}\overset{N}{\underset{i=2}{\sum }}\left(t_{i}-t_{i-1}\right)$$
  where *t*_*i*_ are the time indices of transitions, and *N* is the number of transitions.
  *Interpretation:* Measures temporal stability between state changes.
3. *n*-gram entropy (e.g., 3-gram):
$$H_{n\text{-gram}}=-\overset{M}{\underset{i=1}{\sum }}p_{i}\log p_{i}$$
  where *p*_*i*_ is the probability of the *i*^*th*^ unique *n*-length subsequence in *S*.
4. Synchronization metric (approximate phase-locking value): reflects phase synchronization of state dynamics.
$$\text{PLV}\approx |\frac{1}{T}\overset{T}{\underset{t=1}{\sum }}e^{j\phi_{t}}|$$
  where ϕ_*t*_ is the instantaneous phase of the analytic signal obtained using the Hilbert Transform of *S*.
5. Fractional occupancy derivatives: captures how fractional occupancy varies across states.
$${\text{FO}}_{k}=\frac{1}{T}\overset{T}{\underset{t=1}{\sum }}\delta \left(s_{t}=k\right),\;\Delta {\text{FO}}_{k}=\frac{d}{dk}{\text{FO}}_{k}$$
  where δ is the Kronecker delta function, and ΔFO_*k*_ is the gradient (change) across microstates.
6. Graph-theoretical features: construct a directed graph *G* = (*V, E*) where each node represents a microstate, and each edge (*i*→*j*) is weighted by the number of observed transitions. These features measure topological properties of the transition network among states.
  - Graph density:
    $$D=\frac{\left|E\right|}{K\left(K-1\right)}$$
  - Average degree:
    $$\bar{d}=\frac{1}{K}\underset{v\in V}{\sum }\text{deg}\left(v\right)$$
  - Clustering coefficient:
    $$C=\frac{1}{K}\underset{v\in V}{\sum }c_{v}$$
    where *c*_*v*_ is the local clustering coefficient of node *v*.
7. Cross-frequency coupling (mock feature):
$${\text{CFC}}_{k}\sim U\left(0,1\right)$$
  where CFC_*k*_ is a randomly generated scalar for each state *k*.

In total, 80 microstate-informed features were extracted per microstate. These included 35 temporal features, 20 spectral features, 15 temporal complexity features, and 10 higher-order or graph-based features. The extracted features were carefully selected to capture both the temporal and spectral properties of the underlying brain networks, thereby facilitating a comprehensive representation of the brain's dynamic functional architecture.

### 3.5 Classification and model evaluation

To assess the discriminative power of the extracted multi-domain features, we employed a range of traditional and contemporary classification algorithms. The selected models encompassed diverse learning paradigms, including linear classifiers, tree-based ensembles, support vector machines, and gradient boosting techniques, each offering unique strengths in handling high-dimensional and potentially nonlinear data. For each model, rigorous hyperparameter tuning was conducted to optimize performance, ensuring fair and robust evaluation on the Sheffield EEG dataset.

By leveraging a diverse set of ML models alongside multi-domain microstate-informed features, we ensure that the approach captures both linear and nonlinear patterns associated with ASD related brain dynamics. Moreover, the use of stratified cross-validation and model agnostic interpretability techniques (e.g., SHAP) strengthens the generalizability of the findings, enabling the model to perform robustly across individuals. This methodological framework holds promise for translating EEG-based biomarkers into clinically viable tools for ASD detection and monitoring.

#### 3.5.1 Linear models

Logistic regression (LR) is a fundamental linear model widely used for binary classification tasks. It works by estimating the probability that a given input belongs to a specific class, using a logistic (sigmoid) function applied to a linear combination of input features. In our study, LR served as the baseline classifier due to its simplicity, interpretability, and proven effectiveness with neuroimaging data that exhibit approximately linear separability. We fine-tuned the model by optimizing key hyperparameters, including the regularization strength (C) and the penalty type (L1 or L2), to balance model complexity and prevent overfitting.

In the context of ASD classification, logistic regression offers the advantage of interpretability (Abraham et al., 2014), as the model's coefficients directly reflect the contribution of individual features to the prediction. This makes it particularly useful for identifying potentially meaningful biomarkers. Moreover, its low computational cost makes it scalable for large EEG datasets. However, a major limitation of LR is its reliance on the assumption of linear class boundaries. This constraint can hinder its performance when dealing with the complex, non-linear brain dynamics often observed in neurodevelopmental disorders such as ASD.

#### 3.5.2 Tree based models

Tree-based models such as Decision Trees (DT) and Random Forests (RF) are widely used in neuroimaging and EEG-based classification tasks due to their intuitive structure and capacity to handle non-linear relationships. A Decision Tree classifier works by recursively partitioning the feature space into homogenous subregions using a tree-like structure, where each internal node represents a decision rule on a feature, and each leaf node represents a predicted class label. While simple and interpretable, standalone Decision Trees are prone to overfitting, especially in high-dimensional neurophysiological data.

To address these limitations, Random Forest (RF), an ensemble of multiple Decision Trees, is employed to improve robustness and generalizability. RF constructs numerous trees on bootstrapped samples of the training data and aggregates their predictions (majority voting) to make a final decision (Breiman, 2001). It also introduces feature randomness during tree construction, ensuring decorrelated trees and reducing variance. For ASD classification, RF offers strong performance due to its ability to model complex feature interactions and handle noisy, high-dimensional EEG data. Moreover, RF naturally provides feature importance measures, aiding the interpretability of results, which is an essential requirement in clinical neuroscience.

Despite their strengths, tree-based models may lack fine-grained probabilistic interpretation and can still overfit in small datasets without adequate regularization or pruning strategies.

#### 3.5.3 Support vector machines

Support vector machine (SVM) is a powerful supervised learning algorithm that constructs an optimal hyperplane to separate data points of different classes by maximizing the margin between them (Lotte et al., 2018). In this study, we employed the radial basis function (RBF) kernel to enable the model to capture non-linear decision boundaries, which are particularly relevant in the context of complex neurodevelopmental disorders like ASD. Hyperparameter tuning for SVM involved optimizing the regularization parameter C and kernel coefficient Y. SVM was included in our comparison due to its proven efficacy in EEG-based classification tasks and its ability to handle high-dimensional feature spaces. In ASD research, SVM has shown strong discriminative performance, especially when non-linear relationships among brain dynamics are present. However, the model's reliance on support vectors and kernel transformations can hinder interpretability, making it less transparent in clinical applications compared to simpler linear models.

#### 3.5.4 Neural networks

Artificial neural networks (ANNs) are flexible, non-linear models inspired by biological neural systems that can learn complex mappings between input features and target classes. In this study, we implemented a shallow feedforward neural network architecture to explore its capacity to model the intricate, non-linear dependencies inherent in EEG-derived microstate features. The architecture comprised one or two hidden layers with ReLU activation functions and was trained using the Adam optimizer. Hyperparameter optimization involved tuning the number of hidden units, learning rate, batch size, and dropout rate to prevent overfitting. Neural networks were included in our model comparison due to their proven efficacy in neuroimaging and EEG-based classification tasks. In the context of ASD diagnosis, NNs have the potential to uncover subtle, higher-order interactions among multi-domain EEG features that may be overlooked by linear models (Roy et al., 2019; Bashivan et al., 2016). However, despite their powerful predictive capabilities, neural networks suffer from limited interpretability and often function as black-box models, posing a challenge for clinical adoption where understanding the basis for predictions is crucial.

#### 3.5.5 Boosting algorithms

Boosting algorithms are powerful ML techniques that combine multiple base estimators to improve predictive accuracy. In this study, we focus on prominent gradient boosting algorithms, namely XGBoost and LightGBM. XGBoost (Extreme Gradient Boosting) is a powerful ensemble learning algorithm that builds an additive model in a forward stage-wise manner by optimizing a differentiable loss function. In EEG-based ASD classification, XGBoost has gained traction due to its high predictive performance, scalability, and ability to handle imbalanced datasets and missing values (Heinsfeld et al., 2018). It constructs multiple shallow decision trees where each new tree corrects the residual errors of the previous ones. Hyperparameters such as learning rate, number of estimators, maximum tree depth, subsampling rate, and regularization parameters are typically optimized through cross-validation. XGBoost is particularly effective in capturing complex, non-linear relationships among features derived from EEG signals, including microstate temporal, spectral, and graph-based dynamics. Despite its nonlinearity, it provides feature importance metrics, offering a level of interpretability crucial for understanding the neurophysiological correlates of ASD.

LightGBM, another gradient boosting framework developed by Microsoft, shares the fundamental principles of XGBoost but introduces innovations like histogram-based decision tree learning and leaf-wise growth, enabling faster training on large datasets. It is especially efficient in high-dimensional feature spaces, making it suitable for EEG data where features from multiple domains (temporal, spectral, complexity) are integrated (Ke et al., 2017). LightGBM is known for its low memory footprint and native support for categorical variables, reducing preprocessing complexity. In the context of ASD, LightGBM has been used to capture subtle patterns in neural activity, providing robust classification performance. Although slightly less interpretable than XGBoost due to its aggressive optimization, it supports SHAP-based explanations, allowing insight into how specific EEG-derived features contribute to ASD prediction.

#### 3.5.6 Ensemble techniques

Ensemble learning techniques combine predictions from multiple base learners to enhance classification performance, reduce overfitting, and improve generalizability compared to single models. Among ensemble methods, stacking (stacked generalization) is particularly powerful, as it leverages the strengths of diverse base models by training a meta-learner on their outputs. In this study, we implemented a stacking ensemble in which XGBoost and LightGBM served as the base learners, selected for their superior accuracy in our initial benchmarking. These models were chosen for their complementary strengths, which excel in capturing complex non-linear relationships through gradient boosting (Wolpert, 1992; Breiman, 2001; Chen and Guestrin, 2016). The meta-learner was Logistic Regression, selected for its interpretability and its ability to learn optimal combinations of base-model predictions. This design aimed to achieve high predictive accuracy for ASD classification while maintaining clinical interpretability, an essential consideration in translational neuroscience applications.

#### 3.5.7 Performance evaluation

The classification models were assessed using three primary evaluation metrics: accuracy, sensitivity, and specificity. Accuracy provides an overall measure of performance by quantifying the proportion of correctly classified subjects across all classes. Sensitivity, also referred to as the true positive rate, measures the model's effectiveness in correctly identifying individuals diagnosed with ASD. Conversely, specificity, or the true negative rate, evaluates the model's ability to correctly classify neurotypical (NT) individuals. Together, these metrics offer a balanced view of performance, particularly important in clinical diagnostic contexts where both false positives and false negatives have significant implications (Fawcett, 2006). In addition to accuracy, sensitivity, and specificity, we report the F1 score, which is defined as the harmonic mean of precision and recall. The F1 score provides a balanced summary of classification performance by jointly accounting for false positives and false negatives, making it particularly informative in clinical classification problems such as ASD, where both missed diagnoses and false alarms are consequential. While the F1 score was not used as the primary criterion for model selection, it was included to facilitate holistic comparison across classifiers, alongside accuracy, sensitivity, and specificity.

To facilitate a systematic comparison, the performance outcomes of all classifiers were compiled into a comprehensive results (Table 1). This comparative analysis allows direct evaluation of each model's strengths and weaknesses, supporting the selection of the most suitable classifier for ASD detection. By identifying the model that optimally balances accuracy, sensitivity, and specificity, the analysis ensures that the chosen method not only achieves high overall performance but also maintains diagnostic reliability across both ASD and NT groups.

**Table 1:** Performance comparison of different classifiers for ASD classification.

**Classifier**	**Accuracy (%)**	**Sensitivity (%)**	**Specificity (%)**	**F1 Score (%)**
LR	72.82	85.19	60.55	75.6
RF	79.18	90.24	68.15	81.2
SVM	74.51	90.03	59.03	78.2
NN	76.24	77.24	75.21	76.6
XGBoost	80.87	86.39	75.34	81.8
LightGBM	80.58	87.09	74.09	81.7
Ensemble	80.96	84.88	77.10	81.8

## 4 Results

In this section, we present a comprehensive evaluation of the proposed microstate segmentation framework for ASD classification. The experiments are systematically designed to highlight the effectiveness of microstate-informed analysis in capturing distinctive brain dynamics associated with ASD. To further assess the robustness of our approach, we perform ablation studies across the four feature domains: temporal, spectral, temporal complexity, and higher-order graph-based features. These ablation experiments quantify the individual and combined contributions of each domain toward overall classification performance. Such an evaluation not only validates the utility of microstate segmentation but also provides insights into the relative importance of different neurophysiological feature categories in ASD diagnosis.

To ensure transparency and reproducibility, we provide detailed specifications of our computational environment and implementation framework. All experiments were conducted on a workstation running Windows 11 Pro, equipped with an Intel^®^ Core™ i7-12700H CPU (2.30 GHz), 32 GB of RAM, and an NVIDIA GeForce RTX 3060 Laptop GPU with 3840 CUDA cores and 6 GB of GDDR6 memory. The implementation was carried out primarily in Python (version 3.10), using libraries such as NumPy for numerical computations, Pandas for data manipulation, SciPy for statistical analysis, and NetworkX for graph-theoretical feature extraction. ML experiments used scikit-learn, XGBoost, and LightGBM, while explainability analyses used SHAP. EEG-specific preprocessing and microstate segmentation were implemented using MNE-Python, EEGLAB (MATLAB), and custom scripts. Data visualization was achieved using Matplotlib and Seaborn. Consistent random seeds (random state = 42) were applied across all stochastic processes to guarantee reproducibility of results.

### 4.1 Ablation studies

To systematically assess the individual contributions of each feature domain, we conducted a series of ablation studies. This approach allows us to isolate and evaluate the impact of microstate-informed features across different domains, thereby quantifying their discriminative power and determining the extent to which each domain contributes meaningfully to ASD classification. Our ablation study was structured to begin with microstate-informed temporal features as the baseline domain, and then progressively incorporate additional feature domains. Specifically, we sequentially added microstate-informed spectral features, followed by microstate-informed temporal complexity measures, and finally microstate-informed higher-order metrics. This incremental approach allowed us to assess the individual and cumulative contributions of each feature domain to ASD classification performance.

#### 4.1.1 Cumulative contribution analysis

In this section, we present an in-depth analysis of the cumulative contribution of different microstate-informed feature domains to ASD classification. The objective of this analysis is to understand how the progressive inclusion of diverse feature sets enhances model performance and captures complementary aspects of brain dynamics.

We began with microstate-informed temporal features (TF), including metrics such as mean duration, fractional occupancy, switching rate, dwell entropy, and transition probabilities. These features serve as the foundational representation of temporal dynamics in EEG microstates. Using the XGBoost classifier optimized with hyperparameter tuning, this single-domain feature set achieved an average accuracy of 71.88% (±0.84) across 10-fold cross-validation, establishing the baseline for our cumulative study.

Next, we aggregated microstate-informed spectral features (SF) with TF, adding band-specific power estimates across delta (1–4 Hz), theta (4–8 Hz), alpha (8–13 Hz), beta (13–30 Hz), and gamma (30–45 Hz) ranges for each microstate. These features capture oscillatory dynamics and frequency-specific patterns that complement the temporal representation. The combined TF + SF feature set yielded an improved average accuracy of 78.60% (±1.92) with XGBoost, highlighting the added discriminative power of spectral information in distinguishing ASD from NT subjects.

To further enhance the feature space, we introduced temporal complexity features (TCF), including entropy measures, Lempel-Ziv complexity, and Hurst exponent. These features characterize higher-order structural and complexity aspects of microstate sequences. When combined with TF and SF, the TF + SF + TCF feature set resulted in an average accuracy of 79.24% (±1.81), indicating a meaningful incremental gain attributable to complexity-based metrics.

**Figure 4:**
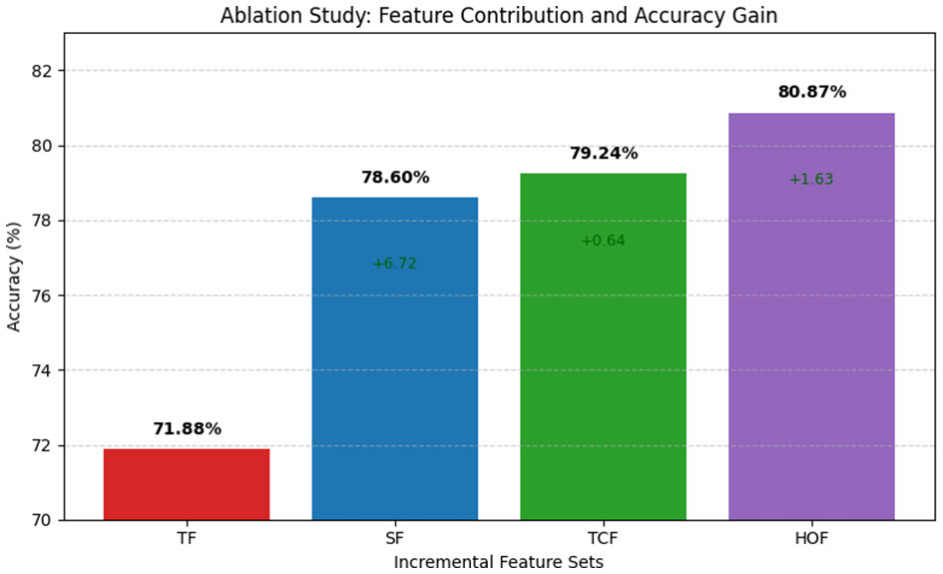
Ablation study results comparing the classification accuracy of four feature sets: temporal features (TF), spatial features (SF), temporal-spatial combined features (TCF), and higher-order features (HOF). Accuracy values are labeled above each bar, with green annotations showing the incremental gain compared to the preceding configuration.

Finally, we integrated higher-order dynamic features (HOF), such as transition entropy, mean inter-transition interval, 3-gram entropy, synchronization metrics (phase-locking estimates), fractional occupancy derivatives, and graph-theoretical measures of microstate transitions. This comprehensive cumulative feature set (TF + SF + TCF + HOF) achieved the highest accuracy of 80.87% (±0.95) in XGBoost, demonstrating that the joint modeling of temporal, spectral, complexity, and higher-order dynamics provides the most robust representation of altered brain network properties in ASD. The contribution of each feature set to the classification performance is illustrated in Figure 4.

### 4.2 Model interpretability and explainable AI analysis

To enhance model interpretability and assess the biological relevance of the extracted features, we performed a comprehensive explainable AI analysis using SHapley Additive exPlanations (SHAP) values (Lundberg and Lee, 2022). This approach quantifies the contribution of each feature to the model's decision-making process, thereby revealing the most influential predictors for ASD classification.

### 4.3 SHAP-based feature importance analysis

We applied SHapley Additive exPlanations (SHAP) analysis to the XGBoost model, as it demonstrated the highest classification performance among all individual models evaluated. SHAP enables the assessment of both local (instance-specific) and global (dataset-level) feature contributions, providing a detailed understanding of how individual features influence ASD classification outcomes.

SHapley Additive exPlanations (SHAP) values quantify the contribution of each feature to the model's prediction by measuring how much that feature shifts the output toward one class or the other relative to a baseline. The mean absolute SHAP value reflects the average magnitude of influence that a feature has on the XGBoost classifier across all subjects. Importantly, SHAP values do not have a fixed upper bound and are dependent on the scale of the model's output; therefore, their magnitude should be interpreted comparatively within the model rather than as an absolute or clinically thresholded measure. For example, a feature with a mean SHAP value of 0.6 indicates a consistently stronger contribution to classification decisions than features with lower values, rather than implying a universal effect size.

**Figure 5:**
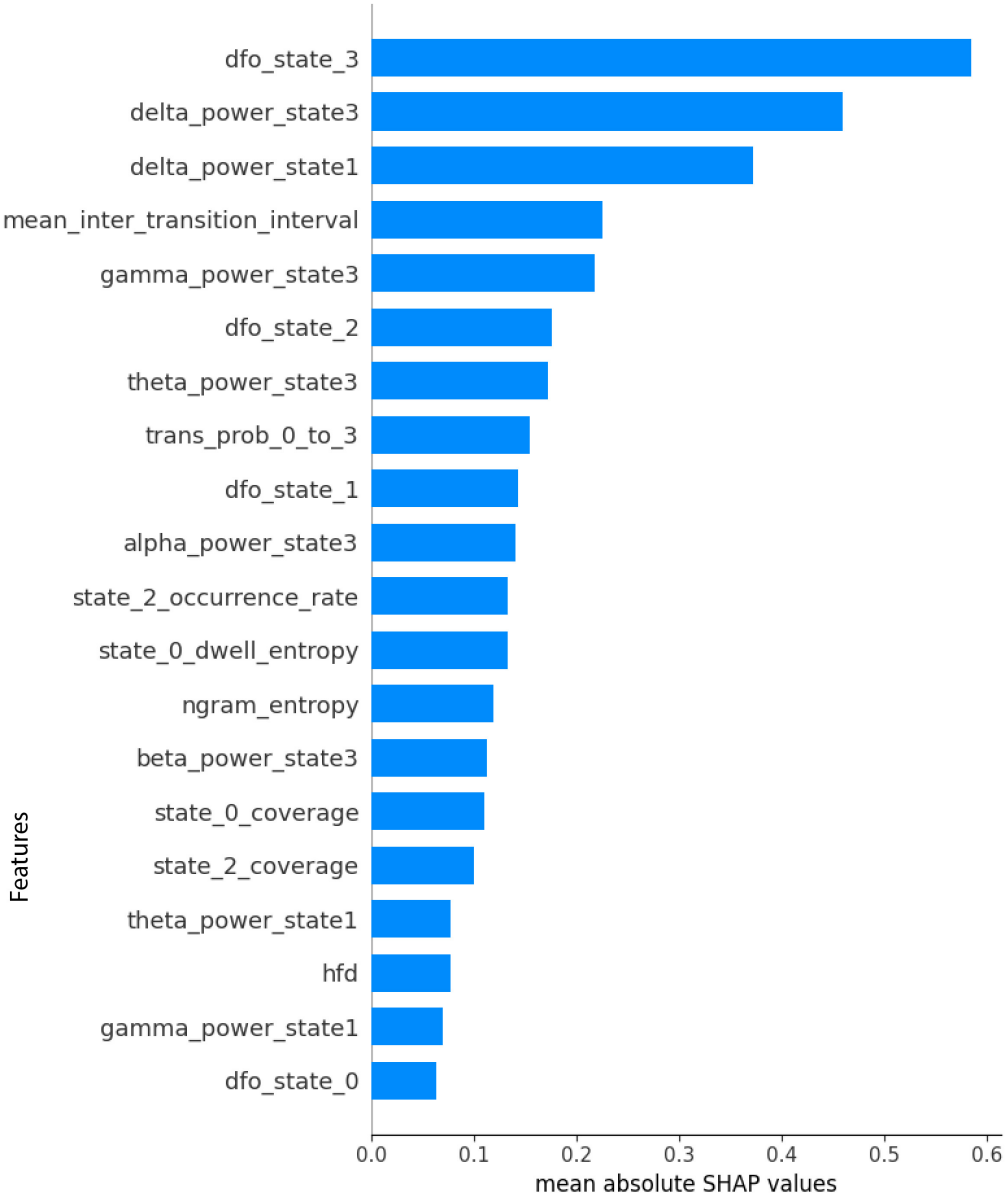
SHAP Feature importance plot where mean absolute SHAP values represent the average magnitude of feature contributions to the XGBoost classifier's predictions for differentiating ASD from NT participants. Features are ranked from highest to lowest importance.

The SHAP analysis identified the top 20 features contributing most significantly to ASD classification, effectively highlighting potential neurophysiological biomarkers. Figure 5 illustrates a clear hierarchy of feature importance, reflecting their relative influence on the model's decision-making process. The most dominant feature was the fractional occupancy derivative of microstate 3, indicating that the classifier relies heavily on the temporal dynamics, specifically, how the proportion of time spent in this microstate changes over time, rather than solely on its absolute occupancy. Following closely in importance were delta power states 3 and 1, both representing delta-band (1–4 Hz) spectral activity during specific microstates. Elevated or altered delta activity within particular states is often linked to cortical slowing and disrupted large-scale network synchronization, phenomena that have been reported in ASD and may reflect underlying neurodevelopmental alterations. The repeated presence of microstate 3 in both temporal and spectral domains suggests that it represents a distinct neural configuration whose dynamic fluctuations and oscillatory characteristics are strongly associated with ASD, positioning it as a promising target for biomarker validation and further investigation.

**Figure 6:**
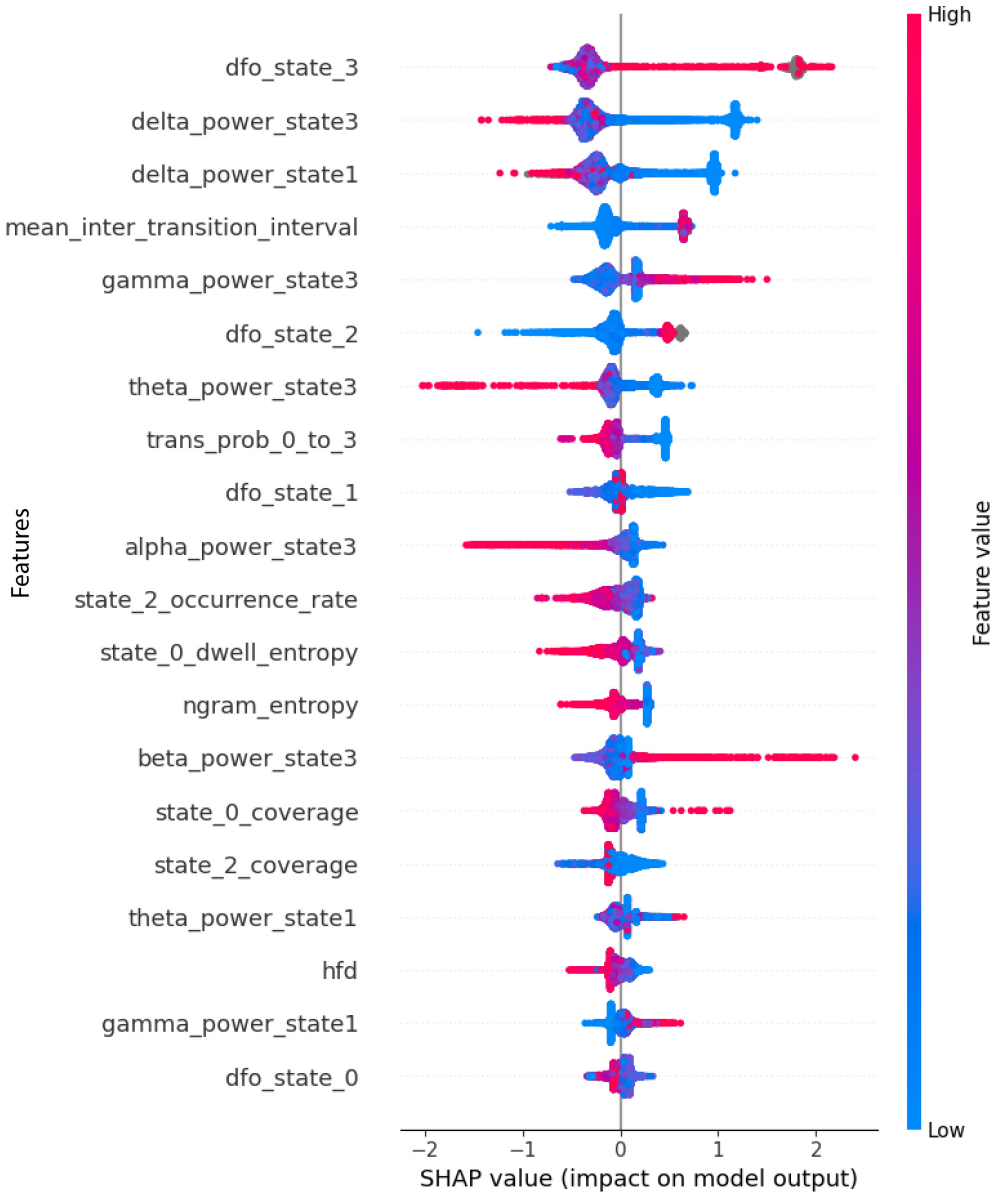
SHAP beeswarm plot showing the top-ranked EEG microstate-informed features contributing to the XGBoost classifier's predictions for distinguishing ASD from NT participants. Each point represents a single subject, with the horizontal axis indicating the SHAP value. Feature values are color-coded from low (blue) to high (red), revealing both the magnitude and direction of each feature's effect on the classification decision.

The SHAP beeswarm plot given in Figure 6 provides a granular view of how each feature influences the XGBoost classifier's decision-making for ASD vs. NT classification. At the top of the hierarchy, the fractional occupancy derivative of microstate 3 (dfo state 3) shows the largest impact, with high values (red) strongly pushing the prediction toward ASD and low values (blue) generally pulling it toward NT. This suggests that rapid temporal fluctuations in the proportion of time spent in microstate 3 are characteristic of ASD, potentially reflecting unstable engagement of a specific large-scale brain network. Similarly, delta power states 3 and 1, representing slow-wave activity in the delta band (1–4 Hz) during specific microstates, exhibit high positive SHAP values when elevated, indicating a strong association between increased delta activity and ASD. This aligns with previous EEG studies linking increases in delta power to cortical slowing and atypical network synchronization in autism (Milne et al., 2019; Duffy and Als, 2012; Sheikhani et al., 2012).

Mid-ranked features further refine this separation. The mean inter-transition interval reveals that shorter or longer periods between microstate changes influence classification in opposite ways; shorter intervals (rapid switching) tend to favor an ASD label, suggesting more fragmented brain-state dynamics. Gamma power state 3 emerges as another strong ASD predictor, with elevated high-frequency (30–45 Hz) activity in microstate 3 likely indicating altered local cortical processing or excitatory inhibitory imbalance. Features such as theta power state 3 and the transition probability from state 0 to state 3 show directional effects, with specific state-to-state transitions or oscillatory activity patterns preferentially occurring in ASD. Fractional occupancy derivatives for other states (e.g., dfo state 2, dfo state 1) also contribute, though less prominently, reflecting that dynamic occupancy changes across multiple states, not just microstate 3, carry discriminative value. Lower-ranked yet meaningful features provide complementary information. Alpha power state 3 and beta power state 3 suggest that oscillatory activity beyond the delta and gamma ranges also plays a role, albeit with a smaller magnitude. Occurrence rate and dwell entropy of certain states capture differences in how frequently states are visited and how variable their durations are, hinting at altered temporal organization in ASD. Complexity measures such as n-gram entropy and Higuchi fractal dimension (HFD) appear lower on the list but still provide marginal gains, potentially capturing nonlinear temporal patterns. The mixture of temporal dynamics, spectral power, transition probabilities, and complexity metrics among the top features underscores that ASD classification is not driven by a single EEG property but rather by a multi-domain interplay of altered brain state stability, oscillatory signatures, and transition structure, with microstate 3 emerging as a central biomarker candidate.

The bidirectional SHAP pattern, where the same feature can exert both positive and negative contributions to ASD classification depending on its value, indicates non-linear, state-dependent alterations in brain dynamics. In certain microstate features, both abnormally high and abnormally low connectivity values may be pathological, suggesting a disruption of the optimal operating range of the corresponding brain networks.

From a dynamic flexibility-vs.-rigidity perspective, metrics such as fractional occupancy derivatives or mean inter-transition intervals reveal that either excessive instability (frequent, rapid switching between states) or excessive rigidity (prolonged persistence in a single state) can signal atypical neural dynamics. Similarly, for spectral characteristics such as delta or gamma power within specific microstates, the observed bidirectionality may imply that atypical connectivity arises across different frequency ranges or amplitude levels, potentially reflecting compensatory adaptations or maladaptive reorganization.

Overall, this pattern reinforces the notion that ASD related brain alterations are not unidirectional but involve deviations in both directions from an optimal balance of network stability, connectivity strength, and oscillatory dynamics.

**Figure 7:**
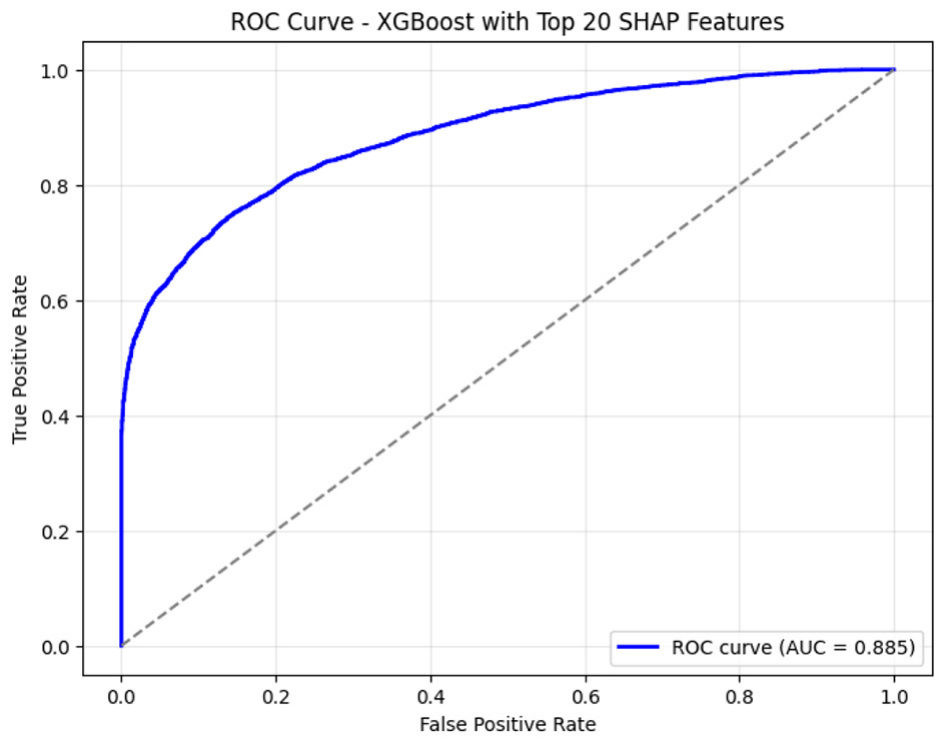
Receiver Operating Characteristic (ROC) curve illustrating performance of the XGBoost classifier using the top 20 SHAP-selected EEG microstate-informed features, with the area under the curve (AUC) indicating overall separability.

To further validate the significance of the features identified by SHAP analysis, we conducted an additional experiment by retraining the XGBoost classifier with only the top 20 SHAP-selected features. These features represent the most influential predictors contributing to ASD classification, as determined by their global importance scores. To ensure optimal model performance and avoid overfitting, we performed extensive hyperparameter tuning using Grid Search with 10-fold Stratified Cross-Validation. This rigorous approach allowed us to identify the best-performing configuration for XGBoost in the reduced-feature-set scenario. The retrained model achieved an impressive accuracy of 80.34%, comparable to that obtained using the full multidomain feature set, demonstrating the robustness and discriminative power of these selected features. Figure 7 illustrates the Receiver Operating Characteristic (ROC) curve for this optimized model, highlighting its ability to achieve a strong balance between sensitivity and specificity in distinguishing ASD from neurotypical subjects.

### 4.4 Statistical validation of SHAP discriminative features

To quantitatively assess the discriminative capacity of the top 20 SHAP-identified EEG microstate-informed features for distinguishing between ASD and neurotypical (NT) participants, we performed a two-step statistical validation process:

1. Non-parametric statistical testing: we applied the Mann–Whitney *U*-test, a rank-based non-parametric test that evaluates whether two independent samples originate from the same distribution. This method is particularly suitable for EEG-derived features, which may violate normality assumptions due to skewness and heavy-tailed distributions. Given two independent groups *X*_1_, *X*_2_, …, *X*_*n*_1__ and *Y*_1_, *Y*_2_, …, *Y*_*n*_2__, the Mann–Whitney *U* statistic is computed as:
$$\begin{array}{l} U=\min\left(U_{1},U_{2}\right),\;U_{1}=n_{1}n_{2}+\frac{n_{1}\left(n_{1}+1\right)}{2}-R_{1}\\ U_{2}=n_{1}n_{2}+\frac{n_{2}\left(n_{2}+1\right)}{2}-R_{2} \end{array}$$
  where *R*_1_ and *R*_2_ denote the sums of ranks assigned to groups 1 and 2, respectively. The null hypothesis *H*_0_ assumes identical distributions for the two groups, while the alternative *H*_*a*_ indicates a shift in location (median difference) between them.
2. Effect size quantification: to complement *p*-values with practical significance, we computed Cohen's *d* and Cliff's Δ for each feature:
  - *Cohen's* *d* quantifies the standardized mean difference between the two groups:
    $$d=\frac{\bar{X}-Ȳ}{s_{\text{pooled}}},\;s_{\text{pooled}}=\sqrt{\frac{\left(n_{1}-1\right)s_{X}^{2}+\left(n_{2}-1\right)s_{Y}^{2}}{n_{1}+n_{2}-2}}$$
    where $\bar{X}$ and Ȳ are the group means, and $s_{X}^{2}$, $s_{Y}^{2}$ are the corresponding sample variances. Positive values indicate higher feature values in ASD, while negative values indicate higher values in NT. Conventional thresholds are small (≈0.2), medium (≈0.5), and large (≥0.8) effects.
  - *Cliff's* Δ is a robust, distribution-free effect size measure representing the probability that a randomly selected value from one group will be larger than a randomly selected value from the other, minus the reverse probability:
    $$\Delta =\frac{\#\left(x>y\right)-\#\left(x<y\right)}{n_{1}n_{2}}$$
    where #(*x*>*y*) counts all pairwise comparisons where an ASD value exceeds an NT value, and #(*x*<*y*) counts the opposite. Values range from −1 (all NT > ASD) to +1 (all ASD > NT), with 0 indicating no difference. Magnitudes can be interpreted as small (≈0.147), medium (≈0.33), and large (≈0.474).

The Mann–Whitney *U*-test identified several features as statistically significant, including delta power state 1, gamma power state 3, and alpha power state 3. This suggests that ASD and NT groups differ consistently in their EEG microstate-related power characteristics and temporal properties. However, significance alone can be misleading without considering effect size, as even small differences can become significant with enough samples or low variance. Therefore, while these features capture genuine group-level differences, their clinical relevance must be validated by effect size metrics.

Features such as n-gram entropy, state 0 dwell entropy, and state 0 coverage exhibit large Cohen's d values, indicating substantial standardized differences between the ASD and NT groups. These metrics primarily represent complexity and stability within microstate sequences, highlighting that ASD brains may exhibit altered dwell patterns and variability in microstate coverage. The large effect size underscores their potential as robust discriminators, even after accounting for variability, making them promising candidates for diagnostic biomarkers.

Cliff's delta flagged features, such as delta power state 1, theta power state 3, and mean inter-transition interval, as having medium-to-large distributional shifts between ASD and NT. This is crucial because nonparametric effect sizes remain robust even under non-normal distributions and in the presence of outliers, which are common in EEG data. These results indicate that state-specific spectral activities and transition dynamics differ consistently between the two groups, reinforcing their physiological plausibility as biomarkers.

**Figure 8:**
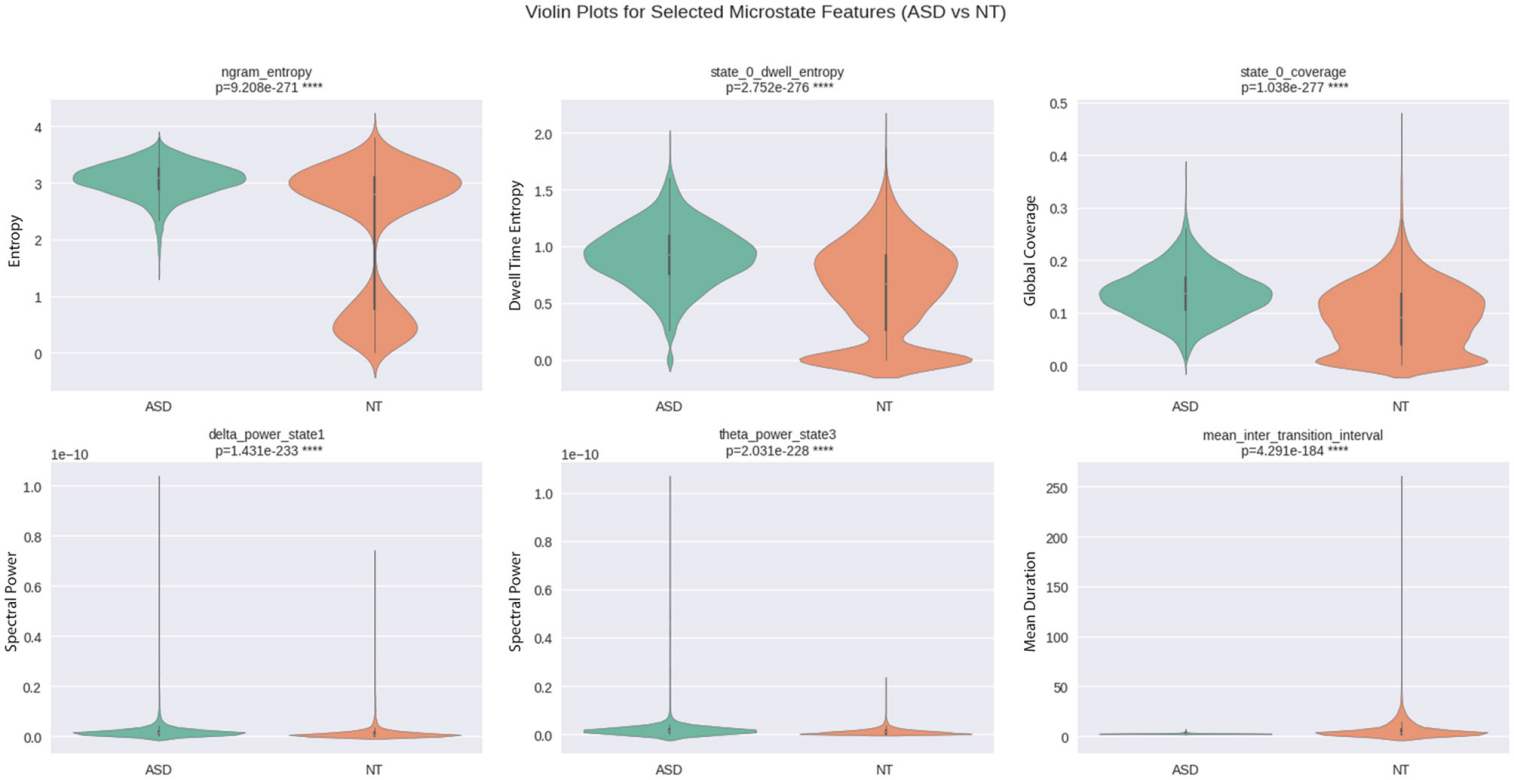
Violin plots showing the distribution of six selected EEG microstate features: ngram entropy, state 0 dwell entropy, state 0 coverage, delta power state1, theta power state3, and mean inter transition interval for ASD and NT groups. Each plot includes the Mann–Whitney *U*-test *p*-value. The violin shape represents the kernel density of the data, and the central marker indicates the median.

As shown in Table 2, the features ngram entropy, state 0 dwell entropy, and state 0 coverage highlight their robustness as candidate biomarkers. These features simultaneously exhibit statistical significance, large standardized differences, and strong distributional separation, suggesting that they reflect profound neurophysiological alterations in ASD. All three relate to microstate temporal organization and sequence complexity, aligning with theories of atypical neural dynamics in autism. Figure 8 presents violin plots illustrating the distribution and statistical significance of the selected features. A substantial overlap exists between ASD and neurotypical distributions for several high-ranking features. This overlap indicates that, while these features capture meaningful group-level differences, they are insufficient for individual-level diagnosis in isolation. Such overlap is expected in heterogeneous neurodevelopmental conditions and underscores the need for multivariate models and cross-diagnostic validation. Their consistency across multiple effect size measures makes them robust candidate features that demonstrate consistent group-level differences and may inform future biomarker development pending validation against other neurodevelopmental conditions.

**Table 2:** Statistical comparison of SHAP selected EEG microstate-informed features using Mann–Whitney *U*-test (*p*-value), Cohen's *d* (standardized effect size), and Cliff's delta (nonparametric effect size).

**Feature**	***p*-value**	**Cohen's *d***	**Cliff's delta**
ngram_entropy	9.21 × 10^−271^	0.999	0.431
state_0_dwell_entropy	2.75 × 10^−276^	0.880	0.435
state_0_coverage	1.04 × 10^−277^	0.825	0.436
delta_power_state1	1.43 × 10^−233^	0.245	0.403
theta_power_state3	2.03 × 10^−228^	0.323	0.394
mean_inter_transition_interval	4.29 × 10^−184^	-0.409	-0.354

## 5 Discussion

### 5.1 Neurobiological interpretation

The discriminative brain network features identified in this study show strong correspondence with established neurobiological models of ASD pathophysiology.

#### 5.1.1 Social-cognition and default mode network (DMN) atypicality

The present analysis revealed a marked reduction in fractional occupancy (FO) for one specific microstate class, accompanied by high discriminative importance of fractional occupancy derivatives (particularly for microstate 3). Furthermore, altered temporal transition dynamics were observed, including significant deviations in specific transition probabilities (e.g., trans prob 0 to 3) and changes in mean inter-transition intervals. These temporal abnormalities were further complemented by elevated sequence entropy measures, such as n-gram entropy, indicating increased unpredictability and variability in the sequence of microstate transitions.

From a neurobiological perspective, this constellation of findings suggests reduced stability and greater temporal volatility in large-scale brain network engagement. Such instability has been widely interpreted as reflecting atypical dynamic coordination between the DMN and attention or executive control networks in individuals with ASD (Padmanabhan et al., 2017; Uddin et al., 2013). The DMN, particularly its posterior-anterior axis, is critically involved in self-referential processing, social cognition, and mentalizing, and disruptions in its temporal organization have been repeatedly implicated in ASD-related deficits in social communication and perspective taking.

If the microstate exhibiting reduced FO corresponds spatially to canonical microstate class C, which has been consistently associated with DMN activity, our results would provide direct electrophysiological evidence for dysregulation of the DMN in ASD. This interpretation aligns with prior EEG microstate and fMRI literature showing diminished DMN stability, atypical DMNsalience/attention switching, and altered integration across social-cognitive networks in autism. To strengthen this inference, a spatial correlation analysis between the observed microstate scalp maps and canonical A-D microstate templates could be performed, thereby confirming the DMN correspondence and reinforcing the link between the observed microstate dynamics and social-cognitive network dysregulation.

#### 5.1.2 Sensorimotor hyper-/hypo-reactivity and motor rigidity

The present findings demonstrated elevated beta-band power within a specific microstate (microstate 3), alongside additional state-specific alterations in delta- and gamma-band power. Notably, these spectral differences were consistently localized to the same microstate, indicating that the observed oscillatory changes are not global but rather tied to a distinct network configuration that recurs over time. Such microstate-specific increases in beta power have been frequently associated with heightened sensorimotor network activation, particularly in fronto-central scalp regions, which are known to subserve motor planning, execution, and sensory gating processes.

From a pathophysiological standpoint, beta band overexpression has been implicated in excessive maintenance of the “status quo” in cortical processing, reflecting a neural predisposition toward motor set maintenance and reduced flexibility in updating motor plans (Spitzer and Haegens, 2017; Engel and Fries, 2010). In ASD, this mechanism is thought to contribute to both motor rigidity and broader cognitive inflexibility, consistent with the restricted and repetitive behavioral patterns characteristic of the condition. Furthermore, atypical beta modulation has been linked to altered sensory reactivity, manifesting as either hyper-responsivity or hypo-responsivity, due to its role in predictive coding and sensory gating.

The concurrent involvement of delta- and gamma-band alterations within the same microstate suggests that the underlying neural network is characterized by a complex interplay between slow oscillatory dynamics (delta) and high-frequency synchronization (gamma), potentially reflecting abnormal integration of bottom-up sensory inputs with top-down motor and executive control. This pattern is congruent with prior EEG and MEG evidence indicating that ASD is associated with both exaggerated and diminished sensorimotor responses, depending on task demands and sensory modality (Marco et al., 2011; Robertson and Baron-Cohen, 2017).

If microstate 3 corresponds to a fronto-central topography typically associated with sensorimotor or salience network activity, the present results may represent electrophysiological evidence for altered state-specific excitability and maladaptive persistence of motor-related cortical assemblies in ASD. These findings thus provide a neural basis for sensorimotor hyper- and hypo-reactivity and motor rigidity, linking the dynamic microstate framework to well-established behavioral and clinical features of autism.

#### 5.1.3 Excitation-inhibition imbalance and neural variability

The analysis revealed increased temporal entropy within microstate 3, accompanied by high discriminative importance of complexity-based metrics, such as Lempel-Ziv complexity and the Higuchi Fractal Dimension (HFD). These measures consistently indicated greater temporal irregularity and reduced predictability in microstate sequence dynamics for individuals with ASD. Importantly, this increased irregularity was not uniform across all brain states but was most pronounced in a specific, recurrent microstate, suggesting that the underlying dysregulation is network-specific rather than global.

When considered alongside the observed increases in gamma- and beta-band power in specific microstates, our results closely align with the excitation-inhibition (E-I) imbalance theory of ASD. This theory proposes that the brain's normal balance between excitatory signals, largely driven by the neurotransmitter glutamate, and inhibitory signals, primarily regulated by GABA, is disrupted in individuals with ASD. Such an imbalance can make brain networks less stable, producing excessive background activity or “neural noise.” As a result, meaningful brain signals become harder to distinguish (lower signal-to-noise ratio), and the precise timing required for different groups of neurons to work together is compromised. In particular, higher levels of gamma activity are thought to reflect too much excitatory activity, while unusual patterns of beta activity may indicate weaker inhibitory control over ongoing brain rhythms. Together, these disturbances may contribute to the atypical brain dynamics often seen in ASD.

The convergence of elevated high-frequency activity and increased temporal complexity in microstate 3 suggests that this state reflects a network prone to excessive excitation, diminished inhibitory regulation, and reduced temporal stability. Such instability may disrupt the network's ability to maintain coherent functional states, leading to aberrant integration of sensory and cognitive information. This mechanistic interpretation aligns with neurophysiological studies reporting elevated trial-to-trial variability, reduced phase synchrony, and altered oscillatory dynamics in ASD across both resting-state and task-based paradigms.

If the spatial configuration of microstate 3 maps onto regions implicated in sensory processing, salience detection, or higher-order integration, the present findings provide further evidence that the E-I imbalance in ASD is not uniformly distributed but preferentially affects specific large-scale brain networks. By linking microstate conditioned spectral and complexity features to the E-I imbalance model, this study offers a fine-grained, temporally resolved electrophysiological perspective on a core pathophysiological mechanism of autism.

#### 5.1.4 Perseveration and cognitive-control switching costs

The temporal dynamics of microstate sequences in the ASD group were characterized by a prolonged mean duration for one microstate class (microstate 2), alongside a reduction in fractional occupancy (FO) for another distinct state (microstate 1). This pattern indicates a tendency for certain brain network configurations to remain active for longer-than-typical periods (“state stickiness”), while other potentially task-relevant or regulatory configurations are under-engaged. In the context of large-scale brain network dynamics, such asymmetry in state engagement suggests an imbalance between stability and flexibility, with the neural system biased toward maintaining a limited subset of network states at the expense of dynamic exploration.

From a cognitive neuroscience perspective, this profile is consistent with behavioral perseveration and increased cognitive control switching costs frequently reported in individuals with ASD. Prolonged occupancy of a given network state may reflect difficulty disengaging from a currently dominant processing mode, while reduced FO in another state may indicate diminished recruitment of networks responsible for cognitive flexibility, attentional reallocation, or adaptive control. These alterations are congruent with previous EEG microstate and fMRI findings showing prolonged dwell times and reduced transition diversity in ASD, both at rest and during cognitive tasks.

Neurobiologically, perseverative tendencies have been linked to atypical interactions among the default mode network (DMN), the salience network (SN), and the executive control network (ECN). If microstate 2 corresponds to a network involved in internally oriented processing (e.g., DMN) or rigid sensorimotor patterns, its prolonged duration could reflect over-engagement of self-referential or repetitive processing loops. Conversely, if microstate 1 maps onto an attentional or salience-related configuration, its reduced FO would be consistent with a diminished capacity to rapidly detect and respond to salient environmental cues, further exacerbating set-shifting difficulties.

Taken together, these findings support the view that ASD is associated with an altered balance between neural persistence and switching, leading to reduced adaptability in cognitive control. By quantifying these dynamics at the millisecond scale, the current study provides electrophysiological evidence linking microstate temporal properties to the well-documented behavioral phenotype of perseveration in autism.

### 5.2 Clinical interpretability

**Figure 9:**
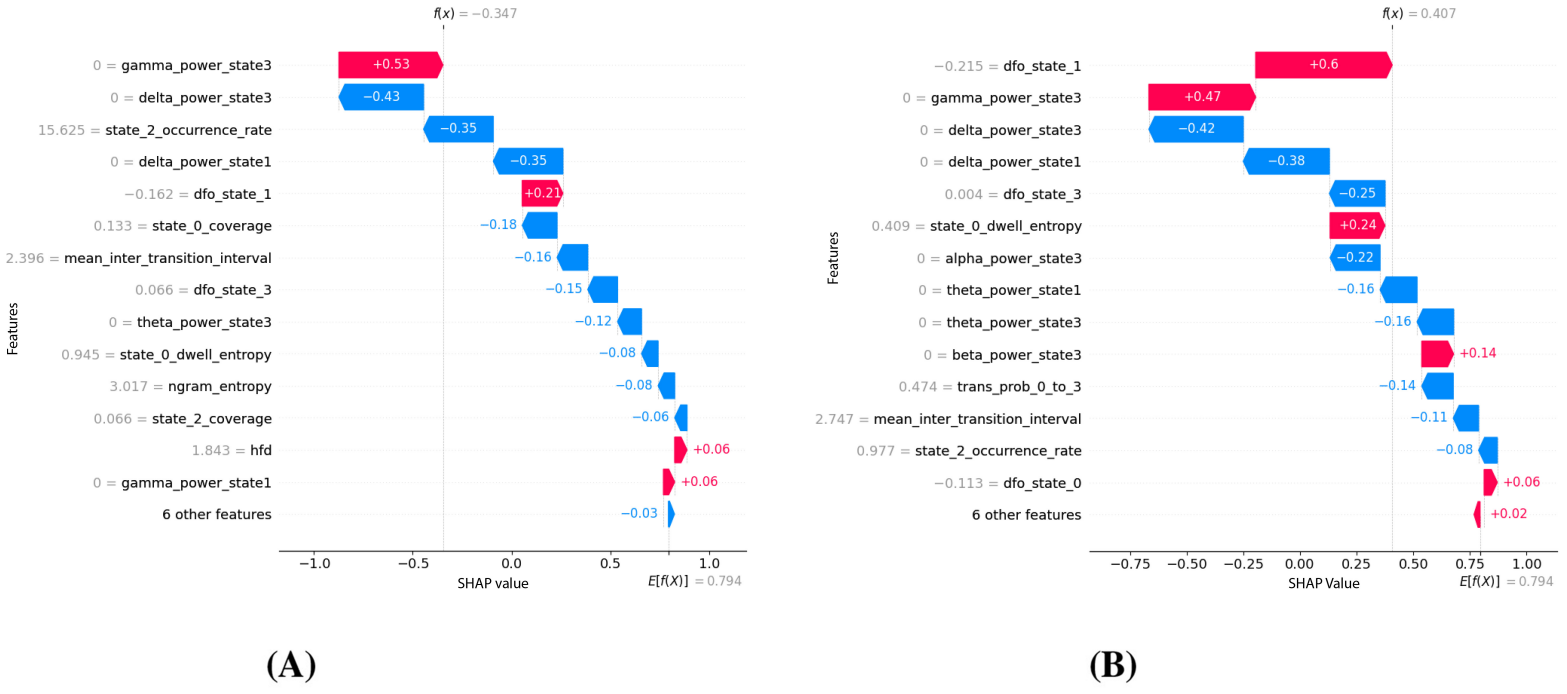
SHAP waterfall plots illustrating subject-specific prediction explanations. **(A)** NT case (Subject 35): regions pushing toward NT classification (negative SHAP values, blue bars) vs. regions pushing toward ASD classification (positive SHAP values, red bars). **(B)** ASD case (Subject 05): the inverse pattern is observed.

The SHAP framework enables clinically interpretable, subject-specific explanations of model predictions by quantifying each feature's contribution to the model's decision. For each individual, these contributions can be visualized using waterfall and force plots, which highlight the brain features that most strongly influence classification toward ASD or NT controls. Figure 9 presents waterfall plots for two representative subjects, illustrating how distinct patterns of brain connectivity drive each classification outcome. Such individual-level interpretability is essential for potential clinical translation, as it allows clinicians to identify the specific neural patterns underpinning a patient's diagnosis. Moreover, the alignment of these explanatory patterns with established ASD neuroimaging findings supports the biological plausibility of the model's decision-making process.

## 6 Limitations

While the proposed microstate-informed multi-domain framework demonstrates promising accuracy and interpretability for ASD classification, several limitations should be acknowledged. First, the study used a publicly available dataset with a relatively modest sample size, which may limit the generalizability of the findings to broader, more heterogeneous ASD populations. Recent empirical work has demonstrated that EEG studies in ASD based on smaller subsamples can systematically overestimate group differences compared to analyses conducted in larger cohorts (Dede et al., 2025), an observation consistent with prior simulation studies highlighting effect size inflation at low sample sizes (Lombardo et al., 2019). Accordingly, the effect sizes reported here should be interpreted as dataset-specific estimates rather than stable population-level parameters. Larger, multi-center datasets are necessary to validate the identified biomarkers across diverse demographic and clinical profiles, including age, sex, and comorbid conditions.

Second, differences in EEG acquisition and preprocessing, such as channel configuration, reference montage, and artifact removal methods, may influence microstate segmentation and subsequent feature extraction. Although data standardization was performed, subtle variations in signal quality and preprocessing pipelines could introduce biases in the learned representations.

Third, the cross-sectional nature of the dataset restricts the ability to assess how microstate dynamics evolve over time or in response to interventions. Longitudinal EEG recordings would enable tracking of developmental trajectories and treatment-related neurophysiological changes in ASD.

Fourth, the study focused on hand-crafted microstate-derived features rather than end-to-end deep representation learning. While this enhances interpretability, it may overlook subtle non-linear patterns that deep neural models could capture. Future work could explore hybrid approaches that combine interpretable microstate features with deep temporal embedding networks.

Finally, although the current model is explainable via SHAP analysis, it requires clinical validation to establish its translational utility. Further testing on real-world clinical EEG data, along with comparisons with behavioral assessment outcomes, is essential to determine its diagnostic reliability and feasibility in medical settings.

## 7 Conclusion and future directions

This study introduces an interpretable and physiologically grounded framework for ASD classification using EEG microstate-informed multidomain features. Unlike conventional EEG-based diagnostic models that focus on isolated spectral or temporal characteristics, the proposed approach integrates temporal, spectral, complexity, and higher-order dynamic features to capture a richer representation of underlying brain activity. This multidomain fusion yielded an average accuracy of 80.87% with the XGBoost classifier, demonstrating substantial improvement over single-domain models and highlighting the synergistic value of cross-domain EEG descriptors.

To ensure interpretability and uncover potential biomarkers, we conducted a SHAP-based feature importance analysis. The top 20 features identified as major contributors included fractional occupancy derivative for microstate 3, delta-band power in microstates 3 and 1, mean inter-transition interval, and gamma power in microstate 3. Retraining XGBoost using only these top-ranked features still achieved 80.24% accuracy, underscoring their predictive strength and clinical relevance. These features suggest that microstate dynamics in ASD exhibit abnormal persistence within certain states and reduced flexibility in switching, indicating an imbalance between neural stability and adaptability mechanisms that may underlie repetitive behaviors and cognitive rigidity observed in ASD.

The interpretation of potential biomarkers indicates disruptions in networks associated with self-referential and social cognitive processing. A notable reduction in the fractional occupancy of a microstate likely connected to the Default Mode Network (DMN), combined with increased transition entropy, suggests reduced temporal stability and abnormal interactions among functional networks. Furthermore, state-specific alterations in delta, beta, and gamma band power suggest an imbalance between excitatory and inhibitory neural processes, accompanied by sustained activation of motor-related networks. These neurophysiological deviations provide a plausible mechanism underlying key ASD characteristics, including motor rigidity, sensory hypersensitivity, and diminished cognitive flexibility.

Statistical validation using Mann–Whitney *U*-tests, Cliff's delta, and Cohen's d confirmed that several SHAP-identified features, such as n-gram entropy, state 0 dwell entropy, and state 0 coverage, exhibit strong statistical significance and large effect sizes. This dual confirmation through both ML and inferential statistics strengthens the case for these features as robust and biologically plausible biomarkers for ASD. From a clinical perspective, these findings represent an important step toward developing objective EEG-based biomarkers that can complement or enhance traditional behavioral assessments. The proposed interpretable framework bridges computational modeling and neurophysiological insight, enabling clinicians to visualize and quantify brain state dynamics in ASD. Such interpretable EEG markers could eventually assist in early detection, personalized intervention planning, and real-time monitoring of therapeutic response, supporting precision psychiatry in neurodevelopmental disorders.

Future research should aim to validate these findings in larger, multi-center datasets, explore age- and severity-specific microstate dynamics, and develop deep learning architectures capable of modeling microstate sequences while maintaining interpretability. Translating these findings into clinical workflows will require the development of automated EEG-based diagnostic software tools and clinician-friendly visualization dashboards. Such translational advancements could enable routine EEG recordings to be used as rapid, low-cost, and interpretable screening instruments for ASD, moving the field closer to objective, neurobiologically informed diagnostic and monitoring systems that support early and individualized care.

## Data Availability

The original contributions presented in the study are included in the article/Supplementary material, further inquiries can be directed to the corresponding author.
