# Supplementary material for: Explainable AI uncovers novel EEG microstate candidate neurophysiological markers for autism spectrum disorder
Source: Front Comput Neurosci. 2026 Feb 4;20:1763727. doi: 10.3389/fncom.2026.1763727 (PMC12913458; doi:10.3389/fncom.2026.1763727)
Supplement: Supplementary file 1 [file Table_1.docx]

**Microstate-Informed Feature Set (80 Features per Microstate)**

**A. Microstate Temporal Features (35 features)**

These features characterize the temporal organization, persistence, and transition behavior of microstates.

*State-wise temporal metrics (4 microstates × 4 features = 16):*

1.Mean duration – State 1

2.Mean duration – State 2

3.Mean duration – State 3

4.Mean duration – State 4

5.Fractional occupancy (coverage) – State 1

6.Fractional occupancy – State 2

7.Fractional occupancy – State 3

8.Fractional occupancy – State 4

9.Occurrence rate – State 1

10.Occurrence rate – State 2

11.Occurrence rate – State 3

12.Occurrence rate – State 4

13.Dwell entropy – State 1

14.Dwell entropy – State 2

15.Dwell entropy – State 3

16.Dwell entropy – State 4

*Transition-related metrics (16):*

17. Transition probability: State 1 → State 1

18. Transition probability: State 1 → State 2

19. Transition probability: State 1 → State 3

20. Transition probability: State 1 → State 4

21.Transition probability: State 2 → State 1

22.Transition probability: State 2 → State 2

23.Transition probability: State 2 → State 3

24.Transition probability: State 2 → State 4

25.Transition probability: State 3 → State 1

26.Transition probability: State 3 → State 2

27.Transition probability: State 3 → State 3

28.Transition probability: State 3 → State 4

29.Transition probability: State 4 → State 1

30.Transition probability: State 4 → State 2

31.Transition probability: State 4 → State 3

32.Transition probability: State 4 → State 4

*Global temporal organization (3):*

33. Switching rate

34. Recurrence rate

35. Determinism

**B. Microstate Spectral Features (20 features)**

These features capture oscillatory dynamics within each microstate.

*Band-limited power per microstate (4 microstates × 5 bands = 20):*

36. Delta power – State 1

37. Theta power – State 1

38. Alpha power – State 1

39. Beta power – State 1

40. Gamma power – State 1

41.Delta power – State 2

42.Theta power – State 2

43.Alpha power – State 2

44.Beta power – State 2

45.Gamma power – State 2

46.Delta power – State 3

47.Theta power – State 3

48.Alpha power – State 3

49.Beta power – State 3

50.Gamma power – State 3

51.Delta power – State 4

52.Theta power – State 4

53.Alpha power – State 4

54.Beta power – State 4

55.Gamma power – State 4

**C. Microstate Temporal Complexity Features (15 features)**

These features quantify irregularity, long-range dependence, and symbolic complexity of microstate sequences.

*Global complexity measures (15):*

56. Sample entropy

57. Permutation entropy

58. Multiscale permutation entropy

59. Lempel–Ziv complexity

60. Detrended fluctuation analysis (DFA exponent)

61. Hurst exponent

62.Sequence entropy

63.Symbolic entropy

64.Shannon entropy of state distribution

65.Entropy rate

66.Complexity index (combined entropy–LZ metric)

67.Temporal unpredictability index

68.Long-range temporal correlation strength

69.Complexity variance across epochs

70.Complexity asymmetry index

**D. Higher-Order / Graph-Based Microstate Features (10 features)**

These features model microstate transitions as a dynamic network.

*Higher-order dynamics and graph metrics (10):*

71. Transition entropy

72. Mean inter-transition interval

73. 3-gram entropy

74. Synchronization metric (phase-locking estimate)

75.Fractional occupancy derivative – State 1

76.Fractional occupancy derivative – State 2

77.Fractional occupancy derivative – State 3

78.Fractional occupancy derivative – State 4

79.Graph density of transition network

80.Average clustering coefficient of transition graph

**Abbreviations**

Abbreviations used in feature labels are defined as follows:

DFO – fractional occupancy derivative; HFD – Higuchi fractal dimension; LZC – Lempel–Ziv complexity; FO – fractional occupancy; MII – mean inter-transition interval; n-gram entropy – higher-order transition entropy. Trans_prob – Transition Probability.

**Dataset Table**

| **Characteristic** | **ASD Group** | **NT Group** |
| --- | --- | --- |
| **Number of participants** | 28 | 28 |
| **Age range (years)** | 18–68 | 18–68 |
| **Mean age (years)** | Not reported | Not reported |
| **Sex distribution (M/F)** | Not reported | Not reported |
| **Recording condition** | Eyes-closed resting state | Eyes-closed resting state |
| **Recording duration** | 150 seconds | 150 seconds |
| **EEG system** | BioSemi ActiveTwo | BioSemi ActiveTwo |
| **Sampling rate** | As provided in dataset | As provided in dataset |
| **Number of channels** | 64 (variable across subjects) | 64 (variable across subjects) |
| **Ethical approval** | \multicolumn{2}{c}{UK Health Research Authority (IRAS ID: 212171)} |  |
|  | **UK Health Research Authority (IRAS ID: 212171)** |  |
